# Positive Economic, Psychosocial, and Physiological Ecologies Predict Brain Structure and Cognitive Performance in 9–10-Year-Old Children

**DOI:** 10.3389/fnhum.2020.578822

**Published:** 2020-10-28

**Authors:** Marybel Robledo Gonzalez, Clare E. Palmer, Kristina A. Uban, Terry L. Jernigan, Wesley K. Thompson, Elizabeth R. Sowell

**Affiliations:** ^1^Children’s Hospital Los Angeles, Los Angeles, CA, United States; ^2^Department of Psychiatry, University of California, San Diego, San Diego, CA, United States; ^3^Center for Human Development, University of California, San Diego, San Diego, CA, United States; ^4^Public Health, University of California, Irvine, Irvine, CA, United States; ^5^Department of Family Medicine and Public Health, Division of Biostatistics, University of California, San Diego, San Diego, CA, United States; ^6^Department of Pediatrics of the Keck School of Medicine, University of Southern California, Los Angeles, CA, United States

**Keywords:** cortical surface area, cognition, SES, poverty, proximal processes, resilience

## Abstract

While low socioeconomic status (SES) introduces risk for developmental outcomes among children, there are an array of proximal processes that determine the ecologies and thus the lived experiences of children. This study examined interrelations between 22 proximal measures in the economic, psychosocial, physiological, and perinatal ecologies of children, in association with brain structure and cognitive performance in a diverse sample of 8,158 9–10-year-old children from the Adolescent Brain Cognitive Development (ABCD) study. SES was measured by the income-to-needs ratio (INR), a measure used by federal poverty guidelines. Within the ABCD study, in what is one of the largest and most diverse cohorts of children studied in the United States, we replicate associations of low SES with lower total cortical surface area and worse cognitive performance. Associations between low SES (<200% INR) and measures of development showed the steepest increases with INR, with apparent increases still visible beyond the level of economic disadvantage in the range of 200–400% INR. Notably, we found three latent factors encompassing positive ecologies for children across the areas of economic, psychosocial, physiological, and perinatal well-being in association with better cognitive performance and the higher total cortical surface area beyond the effects of SES. Specifically, latent factors encompassing youth perceived social support and perinatal well-being were positive predictors of developmental measures for all children, regardless of SES. Further, we found a general latent factor that explained relationships between 20 of the proximal measures and encompassed a joint ecology of higher social and economic resources relative to low adversity across psychosocial, physiological, and perinatal domains. The association between the resource-to-adversity latent factor and cognitive performance was moderated by SES, such that for children in higher SES households, cognitive performance progressively increased with these latent factor scores, while for lower SES, cognitive performance increased only among children with the highest latent factor scores. Our findings suggest that both positive ecologies of increased access to resources and lower adversity are mutually critical for promoting better cognitive development in children from low SES households. Our findings inform future studies aiming to examine positive factors that influence healthier development in children.

## Introduction

According to the Census Bureau for 2017, 38.8% of children in the United States experienced low socioeconomic status (SES), living in households ranging from deep poverty to low-income (Fontenot et al., 2018). SES is most often defined by family income and has been reported widely in association with outcomes in cognitive performance in children, such that children from lower SES backgrounds perform worse compared to peers from more economically advantaged backgrounds (McLoyd, 1998; Evans, 2004; Farah et al., 2006; Noble et al., 2015). Childhood low SES has also been linked to increased risk of emergence of mental and physical health problems in adulthood (Melchior et al., 2007; Jensen et al., 2017). Most recently, studies have reported associations of SES with characteristics of whole-brain and regional structure in children (Hair et al., 2015; Noble et al., 2015; Farah, 2017; McDermott et al., 2019). Several hypotheses about the causal pathways by which SES influences development have been suggested, including: (1) the indirect of effects of SES through exposure to environmental stress that alters neural structures important for stress regulation (the hypothalamic-pituitary-adrenal axis); and (2) the indirect effect of SES through ecologies of resource deprivation such as food and housing insecurity, parental characteristics, cognitive stimulation in the environment, and prenatal care (for reviews see Brito and Noble, 2014; Johnson et al., 2016; Farah, 2017). Thus, SES as measured by family income is a distal measure of the proximal processes underlying risk and resilience in developmental outcomes. Proximal processes in the context of child development have been defined as forms of interactions between the child and environment thought to influence development over time (Bronfenbrenner, 1994). To advance our understanding of the pathways by which SES is associated with brain and cognitive development, we must consider a bio-psycho-social-ecological model that includes an array of proximal processes potentially traveling with low SES and development, to then examine the unique or joint influence they exert on development across the economic spectrum.

Bronfenbrenner’s bio-psycho-social model suggests that development during childhood is dependent on reciprocal interactions that occur within the nested and dynamic environments of children (Bronfenbrenner, 1979; Bronfenbrenner and Morris, 2006). For low SES children, there can be tremendous variability in the quality of these environments (i.e., characteristics of parents, interactions with family, community, school, and neighborhood experiences). These transactional processes form linked social ecologies that jointly shape risk and resilience for development during childhood (Ungar et al., 2013). Low SES has been correlated to various degrees with psychosocial risk (e.g., increased family conflict) and sociodemographic risk (unplanned pregnancies and single parent households), experiences thought to expose children to adversity (Duncan and Brooks-Gunn, 2000; Hussey et al., 2006; Kim et al., 2018). Studies that have examined an array of proximal factors in relation to risk and cognitive development suggest that it is the number of cumulative risk rather than the specific type of risk that best predicts development (Furstenberg et al., 2000; Zigler et al., 2011). However, low SES does not always translate to adversity or material deprivation. In fact, positive factors of social and community support, such as positive parenting and positive school environments, have been associated with better developmental outcomes among children from low SES backgrounds (Benard, 1991; Whittle et al., 2014). Thus, among children growing up in low SES households, investigating how individual differences in their ecologies exacerbate or mitigate the possible negative influences of low SES on cognitive and brain development is complex. To identify promotive factors for developmental outcomes for children in low SES households, it is important to consider an array of proximal measures encompassing the dynamic ecologies for children.

Importantly, when considering the effects of bio-psycho-social-ecological processes on development, we must acknowledge that early experience, such as prenatal exposures and early birth outcomes, influence brain development throughout childhood. For instance, adverse perinatal factors, such as low birth weight (Papadopoulou et al., 2019) and maternal substance use (McLachlan et al., 2016), have also been associated with stress dysregulation in childhood and adolescence. These same adverse perinatal factors are associated with cortical alterations (Hendrickson et al., 2018; Pascoe et al., 2019). Some studies have reported children from low SES backgrounds are at risk for prematurity and low birth weight (Malecki and Demaray, 2006; Kelly and Li, 2019). Despite these connections, proximal measures in the domains of adverse childhood experiences, economic and psychosocial, physiological health, and perinatal exposures have rarely been examined collectively and within a single model as predictors of development (Liaw and Brooks-Gunn, 1994).

Across the SES spectrum, including children from low SES households, there is tremendous variability in the quality and experience of such bio-psycho-social ecologies. Some children from low SES households may likely experience positive ecologies, however, it is unknown to what extent these positive ecologies promote healthier development within a low SES context compared a higher SES context. In this study, we examine the large and diverse typically developing sample of the Adolescent Brain Cognitive Development (ABCD) study, to first test replication of SES associations with developmental measures of cognitive performance and total cortical surface area. We then go beyond associations of SES and leverage the bio-psycho-social-ecological model by utilizing 22 proximal measures of economic, social, physiological, and perinatal ecologies to accelerate our understanding of how these contexts uniquely influence cognitive and brain development. We implemented a Group Factor Analysis (GFA), a multivariate approach which allowed for the examination of relationships among the 22 proximal measures to identify latent factors descriptive of ecologies among children. We hypothesized latent factors for positive bio-psycho-social-ecologies encompassing economic, psychosocial, physiological, and perinatal health would predict better developmental outcomes beyond the variability explained by SES. Further, we considered whether the associations between positive ecologies and developmental measures could be moderated by SES, such that the positive ecologies could relate to the developmental measures differentially for children in low SES households relative to children in higher SES households. Findings from our large sample study can inform future smaller scale studies of risk and resilience for cognitive and brain development with children growing up low SES, as well across the SES spectrum.

## Materials and Methods

### Participants

Data were obtained from the ABCD Study. The ABCD 2.0.1 data release was downloaded from the NIMH Data Archive ABCD Collection (10.15154/1504041) and contained baseline data for a total of *N* = 11,875 children ages 9–10 years old. Demographics of the sample are described in Table 1. Baseline data that passed quality assurance (*N* = 462 excluded) and had complete cases for FreeSurfer imaging data (*N* = 341 missing), demographic measures (household income: *N* = 1,018 missing; Sex: *N* = 4 missing), and the 22 proximal measures, were included in the analyses for a total of *N* = 8,158.

**Table 1:** Distributions for cognition scores, total cortical surface area (SA), age, sex, and race-ethnicity are shown for the overall sample and each income-to-need group as defined by the federal poverty level.

	Deep poverty <50%	Poverty 50– <100%	Near poverty 100– <200%	Mid income 200– <400%	High income ≥400%	Total sample
Cognition scores mean (SD)	78.9 (9.2)	81.5 (8.1)	84.5 (8.6)	87.3 (8.1)	89.9 (7.7)	87.1 (8.8)
Total cortical SA (mm^2^) Mean (SD)	1.77e^5^ (1.78e^4^)	1.82e^5^ (1.79e^4^)	1.83e^5^ (1.72e^4^)	1.87e^5^ (1.78e^4^)	1.9e^5^ (1.76e^4^)	1.9e^5^ (1.8e^4^)
Age Mean (SD)	9.83 (0.61)	9.87 (0.61)	9.92 (0.63)	9.90 (0.63)	9.94 (0.62)	9.91 (0.62)
Sex *N* (%)
Female	303 (49.0)	225 (46.2)	628 (48.4)	981 (48.0)	1,776 (47.8)	3,913 (48.0)
Male	315 (51.0)	262 (53.8)	670 (51.6)	1,061 (52.0)	1,937 (52.1)	4,245 (52.0)
Race-ethnicity *N* (%)
White^a^	92 (14.9)	126 (25.9)	515 (39.7)	1,215 (59.5)	2,772 (74.7)	4,720 (57.9)
Hispanic	178 (28.8)	181 (37.2)	362 (27.9)	433 (21.2)	362 (9.7)	1,516 (18.6)
Black^a^	276 (44.7)	133 (27.3)	266 (20.5)	200 (9.8)	116 (3.1)	991 (12.1)
Asian^a^	4 (0.6)	4 (0.8)	13 (1.0)	18 (0.9)	91 (2.5)	130 (1.6)
Other^a^	68 (11.0)	43 (8.8)	142 (10.9)	176 (8.6)	372 (10.0)	801 (9.8)
Total sample N (%)	618 (7.6)	487 (6.0)	1,298 (15.9)	2,042 (25.0)	3,713 (45.5)	8,158 (100)
U.S. Population >18 years (%)^b^	8.0	9.5	21.3	28.9	32.3	-

*^a^Non-Hispanic; ^b^U.S. Census Bureau, Current Population Survey, 2018 Annual Social and Economic Supplement*.

The recruitment strategy has been described in detail previously (Garavan et al., 2018). Children were recruited from 22 study sites and ABCD is following children at 21 study sites across the United States. A school-based recruitment strategy was developed to achieve a cohort of families that was diverse in income, race-ethnicity, and cultural background and has been described in detail by Garavan et al. (2018). Demographic information for age, sex (female: 1, male: 0), and race-ethnicity were examined. Race-ethnicity was recoded to include five categories: Hispanic, and non-Hispanic White, Black, Asian, and more than one race.

### SES: Income-to-Needs Ratio

SES was estimated using the income-to-needs ratio (INR). The INR was calculated by dividing reported household income by the federal poverty threshold for a given household size. A lower INR ratio indicated higher SES. Gross household income and the number of household members were reported by the participants’ caregiver in the Parent Demographics Survey. Income was reported in bins and was adjusted to the median for each income bin. We used the 2017 federal poverty level for the corresponding household sizes from the poverty guidelines updated periodically in the Federal Register by the United States (U.S.) Department of Health and Human Services under the authority of 42 U.S.C. 9902(2). The federal poverty level (i.e., 100% INR) is the necessary income needed for a family of a given size (e.g., $24,600 for a family of 4) to meet the cost of living, including shelter, food, clothing, transportation, and other necessities and determines eligibility for federal government benefit programs. The federal poverty guidelines also specify a threshold for low SES households (<200% INR) and these are subdivided into: deep poverty (<50% INR), poverty (50–100% INR), and near poverty (100–200% INR).

### Proximal Measures for Bio-Psycho-Social Ecologies

We examined 22 proximal measures thought to encompass bio-psycho-social ecologies hypothesized to be associated with cognitive performance and brain structure based on previous literature (Bronfenbrenner and Morris, 2006; Zigler et al., 2011; Ungar et al., 2013; Pepper and Nettle, 2017; Farah, 2018). We grouped the 22 measures into six groups thought to encompass ecologies of economic, psychosocial, physiological and perinatal ecologies: (1) economic security (i.e., food, housing, bills, and medical); psychosocial ecologies: (2) parental characteristics (i.e., education, dual parent households, parental monitoring, and caregiver warmth); (3) school/community environment; (4) risk for adverse childhood experiences (ACEs); (5) physiological health; and (6) perinatal well-being. Table 2 shows a list of variables examined within each group and detailed descriptions for each variable are available in Supplementary Table 1. Previous studies have shown that measures of economic security often travel together, such as food and housing insecurity (Njai et al., 2017). Economic security was measured by a set of questions that determined food security, housing security, ability to pay bills, and access to medical or dental care. Risk for adverse childhood experiences, including history of traumatic events, family conflict, parent psychopathology have been shown to be correlated (Hussey et al., 2006). Parental psychopathology was the average *z*-score of Adult Self-Report scores and parental history of conduct problems (unable to hold down a job, gets into fights, et cetera). Parental ecologies which are comprised of characteristics of the parent, including parental education, parental monitoring and caregiver acceptance (i.e., warmth and responsiveness) were grouped together, while measures of school and community environments were grouped together, i.e., school engagement, school positive environment and neighborhood safety (Collishaw et al., 2009). Importantly, dual parent household was defined by the study caregiver report of whether he/she had a partner who was involved in at least 40% or more of the daily activities of the child. Highest parental education was from parent report of highest education attained among both caregivers when available. Measures of physiological health, specifically BMIz and sleep hours have been closely liked and were grouped together (Carter et al., 2011; Golley et al., 2013), while measures of perinatal health, including birth weight, prematurity and prenatal drug use have also been closely linked together (Malecki and Demaray, 2006; Kelly and Li, 2019). Body Mass Index *z-scores* (BMIz) were calculated using the SAS Program for the 2000 CDC Growth Charts (Centers for Disease and Control and Prevention n.d.) using height (cm), weight (kg), age, and sex. Two participants with implausible birth weights for gestational age were excluded (>4.98 kg at 35 weeks). For BMIz scores, *N* = 46 participants had implausible scores (>4) and were thus excluded. A detailed description of the ABCD baseline protocol, including a description of the variables used have been reported previously (Barch et al., 2018; Zucker et al., 2018).

**Table 2:** List of grouped measures entered into the Group Factor Analysis (GFA) encompassing economic security, psychosocial ecologies (parental, ACEs, and school/community), physiological and perinatal domains, across parent report and youth (Y) report.

GFA groups	Measures					
Economic	Food security	Housing security		Ability to pay bills		Access to medical/dental
Parental	Parental education	Total caregiver warmth (Y)		Parental monitoring (Y)		Dual parent households
ACEs	Family conflict (Y)	History of a traumatic event		Parent psychopathology
School/community	Neighborhood safety (Y)	Positive school environment (Y)		School engagement (Y)
Physiological	Sleep hours	BMIz
Perinatal	Total prenatal conditions	Planned pregnancy	Maternal age at birth	History of prenatal substance use	Gestational age (weeks)	Birth weight (kg)

### Cognitive Performance

The NIH Toolbox^®^ cognition battery was administered as part of the ABCD study baseline neurocognition protocol (Luciana et al., 2018). The Toolbox^®^ provides composite derived *T*-Scores for each participant, summarizing performance across seven cognitive tasks in the domains of language (reading and vocabulary) and executive function related skills (i.e., working memory, processing speed, cognitive flexibility, episodic memory, attention/inhibition). The composite derived *T-Scores* are fully corrected standardized scores that account for demographic characteristics, including gender, education and race/ethnicity (Luciana et al., 2018). Studies have reported extensively on associations of SES with cognitive skills encompassing both language and executive function domains (Ursache and Noble, 2016; Merz et al., 2019). Thus, the total composite cognition score, which encompasses both language and executive function domains, was used as the summary measure of cognitive performance (Bleck et al., 2013; Gershon et al., 2013).

### Cortical Surface Area

The imaging procedures for ABCD imaging acquisition and preprocessing have been described previously (Hagler et al., 2019). Each site applied a standardized structural magnetic resonance imaging (sMRI) protocol that included a T1 weighted scan among other imaging modalities. All imaging data was processed using FreeSurfer pipelines and procedures by the ABCD Data Informatics and Resource Center. Quality control details are described in depth in Hagler et al. (2019). Briefly, sMRI data underwent distortion and motion correction, and cortical surface area reconstruction was derived using T1 weighted images. Trained technicians manually reviewed sMRI data pre and post processing pipelines to evaluate integrity of the images across five artifact categories: intensity inhomogeneity, underestimation of white matter, pial overestimation, and magnetic susceptibility artifact. Each quality control category was assigned a rating of either absent, mild, moderate, or severe. In addition, the trained quality control technicians assigned an overall quality control score, with a score of 1 indicating a passing score and that the cortical surface area reconstruction as usable, or a score of 0 recommending the data be excluded. A rating of severe on any of the five categories was recommended as exclusionary. Imaging data with an overall quality control score of passing were used.

Cortical surface area, in contrast to other morphological measures of brain structure, increases across cortical regions during childhood, while during early adolescence, regions across the cortex begin to shift to a pattern of reduction (Lebel and Beaulieu, 2011; Raznahan et al., 2011; Wierenga et al., 2014). At age 9–10 years, it was expected there would be relatively low variability in total cortical surface area attributable to age, with higher total cortical surface area thought to reflect a more mature child brain. Thus given that previous studies have reported higher total cortical surface area in association with higher SES across a wider age range of individuals (Noble et al., 2015; McDermott et al., 2019b), we identified total cortical surface area as an appropriate measure to examine associations between proximal measures of children’s ecologies and brain structure at age 9–10 years.

### Group Factor Analysis

A Group Factory Analysis (GFA; Klami et al., 2015) was applied to extract latent factors from our 22 proximal measures. One of the strengths of the GFA approach is that it allows for assignment of variables to a specific group. The GFA approach then accounts for the covariances between variables within each group while identifying orthogonal linear latent factors that encompass relationships across all variables. GFA is similar to a Bayesian exploratory factor analysis, except unique to the GFA approach is the implementation of a structural sparsity prior that allows modeling of the dependencies between groups, where each group contains a set of related variables. Thus the GFA approach is appropriate for examining relations across a set of variables, while accounting for more nuanced relations within each group. All 22 proximal measures were assigned to a category (economic security, parental ecology, school/community environment, ACEs, physiological health, and perinatal well-being) and entered into the GFA (listed in Table 2). To test the stability and robustness of the latent factors, we completed 10 different iterations of the GFA. Robust latent factors were chosen based on latent factor loadings that met a 0.9 correlation threshold across all 10 iterations. Robust factor loadings across all 10 GFA iterations were averaged. Separate robust GFAs were examined in split-half samples to test replication of the latent factor loadings. Robust GFA latent factors accounting for more than 5% of the GFA variance were chosen.

### Analytic Strategy

All statistical analyses were done using open source software from the Comprehensive R Archive Network (version 3.4.4; R Development Core Team, 2018). All R code to replicate the analyses, including the GFA, is available at: https://github.com/ABCD-STUDY/gfa_ses. Generalized Additive Mixed-Effect Models (GAMMs) were fitted using the R-package gamm4 (Wood, 2017) to construct additive mixed-effect models. Continuous measures were standardized to a zero mean and unit variance. All models included demographic covariates of age, sex, race/ethnicity as fixed effects, and random effects of site and family identification. First, using separate mixed effect models, we tested whether cognitive performance and total cortical surface area was each predicted by the INR. Second, results from the GFA were interpreted to identify latent factors that described patterns of relations among measures hypothesized *a priori* as proximal measures of economic, psychosocial, physiological, and perinatal ecologies across the entire economic spectrum. We then tested whether the INR was associated with each latent factor in separate mixed effect models by entering the INR and the demographic covariates, including random effects of site and family, as predictors of each latent factor. Third, we tested the associations between each latent factor and total cortical surface area and cognition scores in models that included the INR as a covariate, as well as the demographic covariates and random effects of site and family. To determine the variance statistically attributable to each latent factor, we examined models in which each latent factor was entered individually as a predictor, including the INR and covariates, for each developmental measure. To then examine the additive variance statistically attributable to all latent factors together, we tested a model in which all latent factors were entered together, including the INR and covariates, as predictors of each developmental measure. Last, we examined interactions between the INR and latent factors on total cortical surface area and total cognition scores using the INR thresholds corresponding to the five income categories for federal guidelines: deep poverty, poverty, near poverty, mid-income, and high-income range. We tested the smooth transformation, which allows for non-linear relationships, of the INR and each latent factor in association with cognitive performance and total cortical surface area using a loglikelihood ratio test with the R “anova” function. Models including the smooth term, compared to models including the linear term, with significant chi-square statistics of *p* < 0.001 were determined to be the best fitting model.

To determine the significance of our models, we compared our full model (predictors + covariates) with a reduced model (covariates only). Reduced GAMMs for each dependent measure, i.e., total cortical surface area and total cognition scores, were constructed with only covariates (fixed effects: age + sex + race-ethnicity; random effects: scanner identification number and family membership). Effect sizes, i.e., the variance statistically attributable by each model, were evaluated as the change in *R^2^* between a full model (predictors + covariates) and a reduced model (covariates only). The significance of the effect size was determined using the log-likelihood test with the R “ANOVA” function, comparing the full model to the reduced model, and the significance of the chi-square statistic was examined. Effect sizes with chi-square statistic values of *p* < 0.001 were determined significant.

### Mass Univariate Effect Size Estimation for Cortical Surface Area

Vertexwise imaging data were obtained from the ABCD 2.0.1 fixed release and was available for 11,536 participants. Imaging data that did not pass quality assurance were excluded from our analyses using the FreeSurfer quality control variable for the ABCD baseline tabulated dataset. A total of 8,158 participants who had complete vertexwise data and complete data on all other behavioral measures were included in the vertexwise surface area analyses. Vertexwise data for all subjects for the surface area were concatenated into matrices in MATLAB R2017a. To measure the vertexwise effects of the INR, we conducted a general linear model at every vertex predicting the INR from the surface area. The following fixed effects were included as covariates of no interest: age, sex, scanner identification number, and race-ethnicity. To determine the vertexwise effects uniquely predicted by each latent factor from the GFA we conducted the same mass univariate vertexwise analysis including additional fixed effects of the INR and the other respective latent factors. To account for the genetic relatedness across the sample, we selected at random only one member from each family to be included in the analysis. This created an *N* of 6,954. All behavioral and imaging variables were standardized with zero mean and unit variance before analysis. Cortical maps were smoothed using a Gaussian kernel of 20 mm full-width half maximum (FWHM) and mapped into standardized spherical atlas space. All estimated effect size maps show the mass univariate standardized beta coefficients. Additional maps were created showing the distribution of mass univariate *p*-values across the scalp adjusted for a false discovery rate (FDR) of 5% using the Benjamini-Hochberg procedure implemented in MATLAB 2017a using the “mafdr” function. All *p-value* maps were thresholded based on an alpha level of adj-*p* < 0.05.

## Results

### SES Associated With Total Cortical Surface Area and Cognition

**Figure 1:**
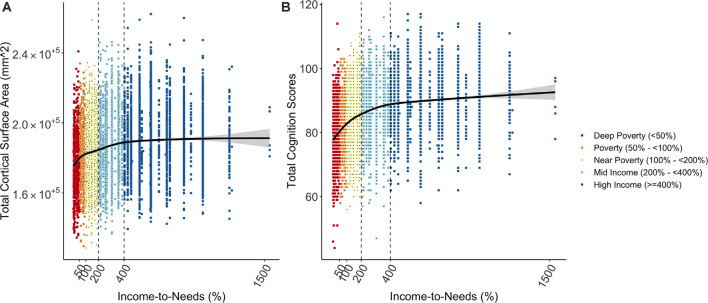
Plots showing the non-linear relationship between socioeconomic status (SES) as measured by the INR **(A)** total cortical surface area and **(B)** total cognition scores. Increases in each developmental measure were steepest for children from lower SES households (near poverty and below, i.e., <200% of the federal poverty level).

In a very large sample of children 9- to 10-years of age from diverse socioeconomic and cultural backgrounds, we tested associations between our SES measure (INR) with developmental outcomes. The smooth transformation, which allows for the modeling of non-linear associations, was the best fit for these associations (cortical surface area: $\chi_{\left(2,N=8,158\right)}^{2}$ = 120.66, *p* < 0.001; cognition: $\chi_{\left(2,N=8,158\right)}^{2}$ = 557.57, *p* < 0.001) and thus the smooth INR term was used in all models. We observed a significant non-linear association between the INR and each developmental measure, such that both total cortical surface area and cognition scores were more strongly related to the INR among children near poverty and below, i.e., <200% of the federal poverty level (Figure 1). The greatest differences in total cortical surface area and cognition scores for the INR appeared to be approximately below 400% of the federal poverty level (i.e., 98,400 for a family of 4), seen clearly in Figure 1. Coefficient values and model fits are shown in Supplementary Table 3 for total cognition scores and Supplementary Table 4 for total cortical surface area.

### Latent Ecologies: Resource-to-Adversity, Social Support, and Perinatal Health

We implemented a (GFA; Klami et al., 2015) to better understand the distinct connections among our 22 proximal measures encompassing economic, psychosocial, physiological, and perinatal ecologies of children. The correlation structure across all 22 measures is provided in Supplementary Figure 1. We found 19 of the proximal measures had significant associations with the INR (Supplementary Figure 2). There was consistent replication across factor loading values for separate GFAs implemented with two split-half samples, for the GFA with a sample with singleton participants only, for the GFA with a sample randomly assigned only one participant per family, and for the GFA implemented with the residuals for each variable after adjusting for fixed covariates (age, sex, and race-ethnicity) and random effects (scanner identification number and family). GFA replications are shown in Supplementary Table 2.

**Figure 2:**
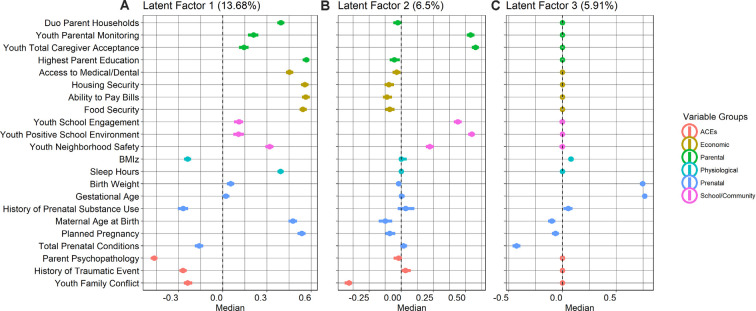
Group factor analysis (GFA) median value loadings (with 95% confidence intervals) for each of the 22 measures for **(A)** latent factor 1: resources-to-adversity (13.68% variance explained); **(B)** latent factor 2: youth perceived social support (6.5% variance explained); and **(C)** latent factor 3: perinatal well-being (5.91% variance explained).

Latent factor 1 (LF1) explained 13.68% of the variance across all proximal measures and described latent ecologies indicative of higher access to social and economic resources, relative to a lower endorsement of adversity across perinatal, psychosocial, and physiological domains (Figure 2). Higher LF1 scores indicated more access to social and economic resources, i.e., food security, ability to pay bills, housing security, access to medical/dental care, higher parental education, dual-parent households, older maternal age at birth, and planned pregnancies. Higher LF1 scores jointly indicated less prenatal conditions and lower endorsement of history of prenatal substance use, suggesting less perinatal adversity from teratogens for *in utero* development. Higher LF1 scores also indicated less exposure to social adversity, i.e., lower ACEs, lower parent psychopathology scores, less endorsement of history of one or more traumatic events, and lower family conflict. Last, higher LF1 scores also jointly indicated less physiological adversity, including sleep hours and lower body-mass-index (BMI) *z-scores*.

Latent factor 2 (LF2) explained 6.5% of the variance across all measures and higher scores indicated more youth perceived social support, loading highly on higher parental monitoring, caregiver acceptance, school engagement, and a more positive school environment, relative to less family conflict. Interestingly, higher LF2 scores, to a moderate extent, also jointly indicated less access to resources, i.e., lower maternal age at birth, unplanned pregnancies, and less endorsement in the ability to pay bills, food, and housing security (Figure 2). Latent factor 3 (LF3) explained 5.91% of the variance and described indices of perinatal health, with higher LF3 scores indicating higher birth weight and longer gestational age, relative to lower total prenatal conditions (Figure 2).

### Latent Ecologies Positively Associated With Total Cortical Surface Area and Total Cognition Scores

**Figure 3:**
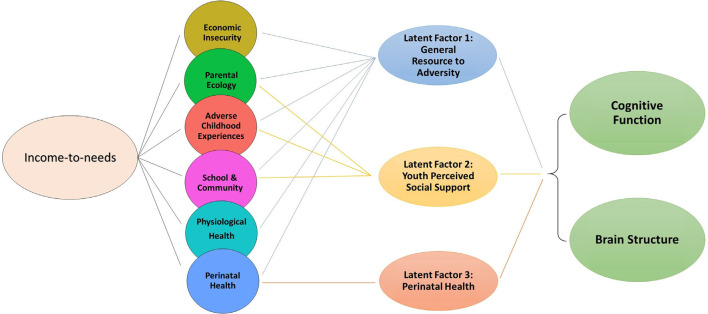
Conceptual model showing that while the INR is associated with proximal measures across ecologies of economic, psychosocial, physiological, and perinatal well-being, three latent factors were derived from the proximal measures to examine associations between each latent factor and cognition scores and total cortical surface area, beyond the INR.

**Figure 4:**
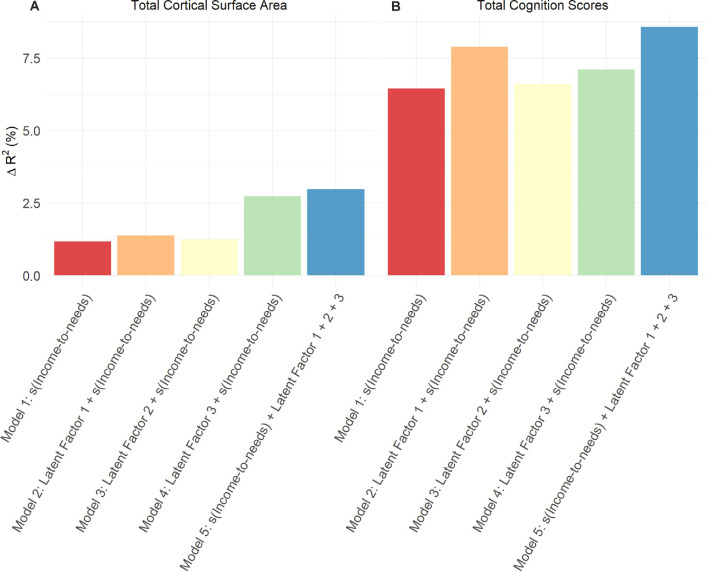
For each developmental measure **(A)** total cortical surface area and **(B)** total cognition scores, effect sizes are shown as the percent of variance statistically attributable to the INR only, each latent factor, and to the additive effect of the INR and all latent factors combined. Change in adjusted *R*^2^ was calculated by comparing each separate model to the null model (fixed effects of covariates and random effects only).

Figure 3 shows the conceptual model in which latent factors were derived from proximal measures hypothesized to be related to the INR, to then examine the unique associations between these latent factors with total cognition scores and total cortical surface area. Among the three latent factors, LF1 (resource-to-adversity), was strongly and positively associated with the INR (*F* = 399.7, edf = 7.18, *p* < 0.001), families with higher economic advantage had more access to resources relative to less adversity across psychosocial, physiological, and perinatal ecologies. The INR was not significantly associated with LF2 of youth perceived social support (*p* = 0.97), nor for LF3 for perinatal well-being (*p* = 0.90). For the association between LF1 and total cognition scores, the smooth term of the LF1 was the best fit ($X_{\left(2,N=8,158\right)}^{2}$ = 18.4, *p* < 0.001). In models adjusting for s(INR), fixed covariates of age, sex, parent-reported race-ethnicity, and random effects of scanner identification number and family, each latent factor was positively associated with both total cognition scores and total cortical surface area such that increases in each latent factor score predicted higher total cognition scores and higher total cortical surface area (Table 3). Associations between each latent factor and the developmental measure were consistent for models in which each latent factor was entered individually, including the INR and covariates as predictors, as well as in models in which all three latent factors were entered together as predictors, including the INR and covariates. Summary coefficients for the full model that included all three latent factors together as predictors, controlling for the INR and covariates, are shown in Table 3. Detailed model results for each model tested, including coefficient and confidence intervals, are shown in Supplementary Table 3 for total cognition scores and Supplementary Table 4 for total cortical surface area. The individual variance statistically attributable to each latent factor (Models 2, 3, and 4) and the additive variance of all latent factors in comparison to the INR (Model 5) are shown in Figure 4. Importantly, these associations were significant when including INR in the models, which demonstrates that variability in individual differences in the developmental measures was statistically attributable to these proximal measures above and beyond SES. In a *post hoc* analysis, we examined individual associations of each proximal measure with both total cognition scores and total cortical surface area to evaluate the contribution of each measure. We found that proximal measures encompassing the latent factors, including measures of economic security, parental ecologies (i.e., parent highest education and psychopathology), and ACEs (i.e., family conflict) were associated with total cognition scores, while similarly, economic security, parental ecologies, and importantly, perinatal heath were associated with total cortical surface area (Supplementary Figure 3). These patterns of the strength of these *post hoc* associations were consistent with the magnitude of the loadings of the proximal measures within each latent factor.

**Table 3:** Standard beta coefficient values and 95% confidence intervals are shown for each latent factor, while for the smooth terms of the INR and LF1 (cognition model only) the *F(edf)* values are shown, for models in which the INR and latent factors were entered together as predictors of total cognition scores and total cortical surface area. The additional variance statistically attributable to this full model compared to a reduced model (covariates only) is shown as *ΔR*^2^.

	Total cognition	Total cortical surface area
*R*^2^	0.30	0.30
Δ*R*^2^_(*Full— Reduced*)_	0.09	0.03
Chi-square	740.7**	282.5**
s(INR)	36.9 (6.18)^±^	13.91 (3.10)^±^
LF 1: Resource-to-adversity	38.6 (3.4)^±^	0.081 (0.055, 0.107)
LF 2: Youth perceived social support	0.042 (0.02, 0.063)	0.027 (0.007, 0.047)
LF 3: Perinatal health	0.073 (0.051, 0.096)	0.121 (0.099, 0.143)

**p < 0.001. ^±^F (edf) statistics for smooth terms.

### SES Moderated Associations Between Latent Resource-to-Adversity and Cognitive Performance

**Figure 5:**
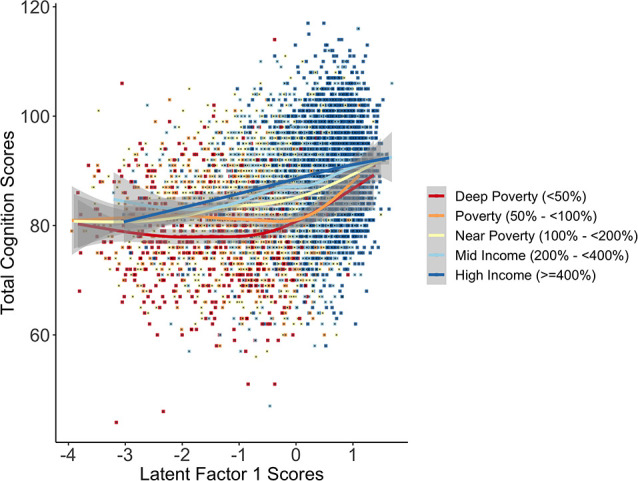
The plot of the interaction of the INR by LF1 scores in association with total cognition scores shows differences in total cognition scores between income-to-need groups varied as a function of LF1 scores. While total cognition scores steadily increased with higher LF1 scores for children with higher SES (mid to high income), total cognition scores for children in poverty and deep poverty showed a protracted shift in scores, revealing an advantage in total cognition scores for children from higher SES households in the middle-range of LF1 scores. Importantly, the gap in total cognition scores between low SES and higher SES narrowed for children at two intersections with latent factor scores: those with higher (approx. +2) scores (i.e., a higher endorsement of access to resources relative to the lower endorsement of adversity) and those with lower (approx. −3) scores (i.e., fewer resources relative to higher adversity).

To determine if there were interactions between SES and the latent factors predicting total cortical surface area and total cognition scores, we generated a grouped INR variable based on U.S. federal guidelines for poverty levels (deep poverty: <50%; poverty: 50–<100%; near poverty: 100–<200%; mid-income: 200–<400%; higher-income: ≥400%). There was a significant interaction of the INR by LF1 scores on total cognition scores such that the association between LF1 and cognition scores differed for deep poverty and poverty compared to higher income groups (deep poverty: *F* = 6.86 (3.1), *p* < 0.001; near poverty: *F* = 10.2 (2.4), *p* < 0.001; near poverty: *F* = 2.0 (2.3), *p* = 0.18; mid-income: *F* = 1.8 (4.2), *p* = 0.16). To interpret the interaction, we plotted LF1 scores predicting total cognition scores by INR groups (Figure 5). The interaction plot shows that among children with high SES, cognitive performance increased steadily with LF1 scores, while for children from low SES households, cognitive performance showed a protracted increase with LF1 scores such that cognitive performance was comparable to their higher-income peers only at the highest LF1 scores. This suggests that for children in the lowest SES households (ranging from poverty to deep poverty), having both increased access to resources and lower endorsement of psychosocial, physiological, and perinatal adversity could be joint and equally promotive ecologies for cognitive performance. There was no significant interaction for LF1 with the INR groups on total cortical surface area ($\chi_{\left(4,N=8,158\right)}^{2}$ = 3.76, *p* = 0.44), nor any significant interactions of LF2 or LF3 with the INR groups on total cortical surface area ($\chi_{\left(4,N=8,158\right)}^{2}$ < = 5.66, *p*s > 0.22) or on total cognition scores ($\chi_{\left(4,N=8,158\right)}^{2}$) < = 4.13, *p*s > 0.39).

### Cortical Surface Area Effect Size Maps

**Figure 6:**
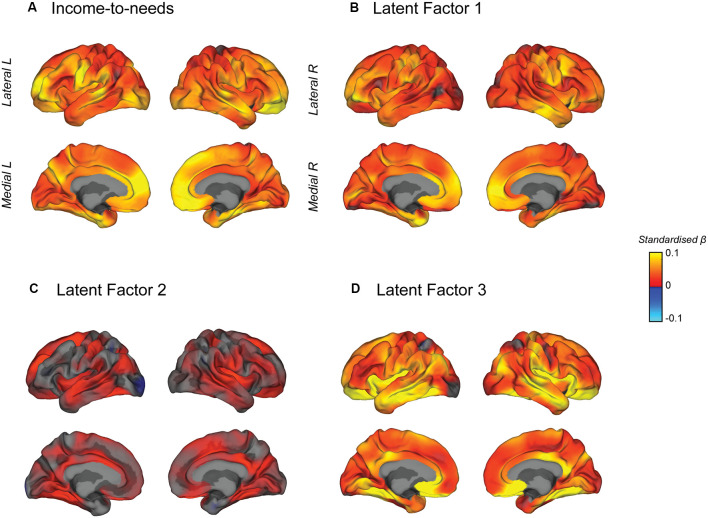
Mass univariate vertex-wise estimated effect size maps predicting surface area from each independent variable, **(A)** the INR, **(B)** latent factor 1, **(C)** latent factor 2, and **(D)** latent factor 3, were created using general linear models at each vertex controlling for age, sex, race/ethnicity, and scanner ID. Maps b–d also included the INR and the other latent factors as additional covariates such that these maps show the unique contribution of each latent factor predicting surface area. The maps show unthresholded standardized beta coefficients. All of the independent variables showed positive effects with the surface area.

A vertex-wise mass univariate analysis across the surface of the cortex was conducted to visualize the effect of the INR and each of the latent factors on surface area (Figure 6). Figure 6A shows the vertex-wise association between the INR (non-transformed) and surface area. Figures 6B–D show the vertex-wise association between each latent factor and surface area (in separate models) all including the INR and the other latent factors as covariates. They, therefore, display the unique variance in the surface area predicted by each latent factor independent of the INR and the other orthogonal latent factors. The maximum vertex-wise beta coefficients for each predictor were *β* = 0.10 for the INR, *β* = 0.093 for LF1, *β* = 0.051 for LF2 and *β* = 0.16 for LF3. INR, LF1, and LF3 showed significant distributed effects across the cortex. INR and LF1 showed very similar effect size maps with the strongest associations on the medial frontal surface, although there is no evidence for strong localization effects. LF3 showed a unique pattern of effects, not explained by INR or the other LFs, with the largest effects in the medial orbitofrontal, fusiform and insular cortices as well as along the temporal lobe. Given that these LFs were included in the same model, this suggests that the associations between these LFs and total surface area may have been driven by effects in different regions of the brain; however, we did not conduct any statistical analyses to specifically test for differences in the localization of effects across these measures. Interestingly, despite being significantly associated with total SA, LF2 did not show any significant vertexwise effects when the other LFs were included in the model, which suggests that LF2 was not associated with any unique variability in cortical SA above and beyond that already explained by the other LFs and INR, which were included in the model. Maps with thresholded vertexwise *p*-values, adjusted for a FDR of 5% are shown in Supplementary Figure 4.

## Discussion

SES has long been known to impact cognitive development and school performance, with more recent research relating to low SES with differences in brain structure thought to reflect a negative effect on brain development (Hair et al., 2015; Noble et al., 2015; Lawson et al., 2017). In this larger and more diverse cohort of children, we replicated previous findings of a continuous association between the INR and developmental measures, with the strongest associations among children from low SES households (Hair et al., 2015; Noble et al., 2015). Methodologically, this study advanced our understanding of the associations between proximal measures of the ecologies of children and cognitive and brain development by: (a) utilizing a large demographically diverse cohort; (b) utilizing proximal measures of the environment of children that more closely reflect the lived experiences of participants; and (c) expanding the scope of developmental ecologies integrated with measures of cognitive and brain development. Within the expanded scope of the 22 proximal measures examined, a GFA identified three latent factors that overall explained 26% of the variability across these measures among individuals aged 9–10 years. The three latent factors were strongly driven by distinct sub-groupings of proximal measures, the first generally encompassing higher access to resources relative to lower adversity in the areas of economic, psychosocial, physiological, and perinatal health, the second youth perceived social support, and the third perinatal well-being. The three latent factors each positive predicted cognitive performance and total cortical surface area at ages 9–10 years beyond economic advantage.

While studies in children often index SES using measures of family income, each proximal measure studied here is thought to represent components of economic, psychosocial, physiological, and perinatal ecologies that are often studied in isolation in association with development (Braveman et al., 2005). Given the complexity of the relations among measures of risk and resilience for children grouping up low SES, it has been difficult to understand how such associations between various economic, psychosocial, physiological, and perinatal factors contribute individually or multiplicatively in explaining differences in developmental outcomes (Guo and Mullan Harris, 2000; Hackman and Farah, 2009; Whittle et al., 2017). Here, using a multidimensional analysis, we identified three latent factors that each describe key relationships between 22 proximal measures that encompass distinct ecologies of the lived experiences of children, with each contributing positively to developmental outcomes. Specifically, LF1 shows interrelations between 20 of the proximal measures such that higher LF1 scores indicate more access to social and economic resources (i.e., higher parental education, economic security, higher maternal age at birth, planned pregnancy, dual-parent households) relative to lower adversity for psychosocial, physiological and perinatal health (lower prenatal substance exposures, and less prenatal conditions, lower family conflict, lower endorsement of traumatic events, lower parent psychopathology, more parental monitoring, safer neighborhood, and lower BMI and better sleep). Higher LF1 scores predicter better cognitive performance and higher total cortical surface area, suggesting that more access to resources relative to lower perinatal, psychosocial and physiological adversity was associated with better cognitive performance across the INR spectrum. Although, LF1 scores did benefit higher-income families more strongly than lower-income families, children from lower income households with the highest LF1 scores showed comparable cognitive performance to their higher-income peers. This suggests that having high access to resources and low exposure to perinatal, psychosocial and physiological adversity were optimal ecological environments that contributed to better cognitive performance for children in lower-income households (ranging from deep poverty to near-poverty). Economic deprivation and psychosocial adversity have been previously described in the literature as poverty-related stress and have been associated with poor mental and physical health (Wadsworth and Berger, 2006; Wadsworth et al., 2008). Thus poverty-related stress may be one overt-stress pathway by which SES has an indirect negative effect on development in children. Also, low SES increases exposure to other risk factors (not captured by LF1 scores, i.e., more pollution and environmental toxins), and it is plausible that these risk factors may exacerbate the negative impact of low-SES on development and attenuate the benefit to cognitive performance and brain development, in particular among children in lower-income households with moderate to low latent factor 1 scores. Increasing resources relative to decreasing adversity, as suggested by LF1, may be important ecologies that promote healthier cognitive outcomes among youth and especially among youth with poverty/deep poverty. This is especially critical for low SES youth, who were more likely to have less resources relative to higher adversity (i.e., lower LF1 scores), and highlights the need to implement public policies that target systemic inequities for youth in poverty/deep poverty by increasing resources and decreasing adversity to promote healthier cognitive outcomes.

While the associations between LF2, youth perceived social support, and each developmental measure were moderate, our findings suggest that having a positive family and community environment is associated with positive developmental outcomes, even though co-occurring with other risk factors, i.e., young maternal age at birth, unplanned pregnancies, lower endorsement of ability to pay bills and food and housing security. Given that LF2 did not show specific associations with the INR, this suggests that higher LF2 or increased youth perceived social support can benefit all children regardless of economic status. LF3 loaded most strongly on perinatal factors that align with the concept of developmental origins of health and disease (DOHaD), which postulate that birth factors (e.g., shorter gestational age, lower birth weight, and more prenatal conditions) are both an outcome and predictor of health and disease (Silveira et al., 2007). Specifically, several prenatal adversities can result in this collection of birth outcomes, and subsequently, this collection of birth outcomes is predictive of increased risk for a sequela of disease outcomes in adulthood. Importantly, we found that LF3 scores benefit children the same across the economic spectrum, suggesting that there was no specific risk captured by the proximal measures in LF3 for children from lower or higher SES.

Previous studies reporting on the association between family income and cortical surface area have suggested the effects of SES on brain structure are stronger in specific regions (Hair et al., 2015; Noble et al., 2015; Kim et al., 2018; McDermott et al., 2019). However, in the present study, with increased sample size and power for detection, we found that the vertex-wise cortical surface area associations for the INR appeared continuous and distributed across the cortex, and while the strongest associations were on the medial frontal surface, we did not find evidence for strong localization effects. From a developmental perspective, the whole-brain cortical surface area increases throughout childhood and begins to show regional decreases in early adolescence (Raznahan et al., 2011; Wierenga et al., 2014). We examined a narrow age group of 9–10 years of age, and it is plausible that associations between SES and regional specificity in brain structure may change with developmental age (Noble et al., 2012; Piccolo et al., 2016; Farah, 2018). Differences in patterns of associations across regions in the brain between the latent factors encompassing distinct aspects of bio-social-ecological systems would be suggestive of differences in underlying mechanisms by which the INR and the latent factors associated with the total cortical surface area. Interestingly, we found that the distribution of effect sizes across the cortex for surface area appeared to be most similar between the INR and LF1 (resource-to-adversity), suggesting that there may be shared pathways by which these ecologies are associated with the cortical surface area. Some studies have suggested regional specificity of the effects of SES in limbic brain structures, including frontal lobe regions, the amygdala, and hippocampus (for a review see Farah, 2017). Our findings suggest that the effect of SES as measured by the INR is much more distributed across the brain for the cortical surface area at age 9–10 years of age. While the visual comparison of the effect size maps between the INR and LF3 (perinatal well-being) shows an apparent qualitative difference, we cannot infer regional differences from these *post hoc* exploratory effect size maps. Instead, these qualitative differences between these maps can guide future studies and hypotheses about the differential pathways by which these ecologies associate with the regional cortical surface area. Also, future studies can examine whether the distribution of SES effects on cortical surface area changes over time during adolescence and whether they shift from global distribution to a pattern that suggests regional specificity.

Further, while we cannot infer any causality or directionality from the observational associations reported here, we found LF1 captured specific relationships between the proximal measures that were indicative of higher access to social and economic resources and lower exposure to adversity, and this was closely linked with SES. This suggests that in our large and diverse sample, increases in family income, in general, were associated with increases in access to resources and decreases in exposure to adversity. Previous studies have attempted small scale interventions in which the household income of families is supplemented and found relative improvement in the allocation and use of economic resources (Rojas et al., 2020; for a review see Barrientos and DeJong, 2006). We found that income was closely tied to other proximal measures that also showed their unique associations with measures of development. While supplementing the income for low-SES households may improve economic resources, it is difficult to know whether this would also generalize to positive changes across other joint social processes that also influence development, i.e., decreased exposure to social and environmental adversity, as well as to better outcomes in development.

## Limitations and Strengths

Understanding the proximal measures that describe the ecology of a child’s environment is important for the investigation of developmental outcomes, as they better assess the compilation of the common daily experience influenced by economic status and subsequently impact development. To best inform interventions or policy reform, we need to better capture the most prominent constellations of experiences that drive brain and cognitive development. To this end, we believe the present findings serve only as an intermediate step towards understanding the incredibly rich proximal and proximal factors that shape America’s youth, as our proximal measures only begin to push the needle towards diving deeper into patterns of daily experiences important for adolescent development. Future studies should strive to capture measures that are even more proximal, and that would ultimately make the present proximal measures appear distal in comparison.

In this study, we have only examined the total cortical surface area as one measure of maturation of brain structure. There are various other measures of brain development that we do not examine that have been reported in association with SES, including cortical thickness, cortical volume, and white matter microstructure (for a review see Farah, 2018). The age at which surface area peaks during childhood is uncertain and studies suggest that there is likely individual variability in the chronological age at which surface area peaks (Raznahan et al., 2011; Shaw et al., 2012; Wierenga et al., 2014; Jernigan et al., 2016). While there were no significant effects of age on the total cortical surface area in our narrow age range of 9–10 year-olds, our cross-sectional analysis cannot determine whether the total cortical surface area in our cohort has yet peaked at age 9–10 years of age. Future longitudinal studies can examine age-related changes in total surface area in association with SES. Further, the relative effect sizes are small, although this is perhaps to be expected from studies examining behavioral and brain outcomes with large samples, given the heterogeneity in individual differences in the population being studied as well as the wide range of factors that could influence development.

Although the composition of the study sample analyzed is overrepresented in the number of households in the higher income range relative to the population income distribution in the United States, our study sample includes a larger representation of children from low SES households than previous studies (Compton et al., 2019). The duration and extent under which children in this cohort have experienced economic and social adversity during their early childhood are not yet known. While it is challenging to differentiate between transitory poverty and chronic poverty, previous literature suggests that even children who have experienced transitory poverty have poorer outcomes compared to children who never experienced poverty (Smith et al., 1995; Duncan and Brooks-Gunn, 2000). Many other risk factors are closely related to low SES not directly examined in this study, such as the child’s mental health and environmental toxins (Evans, 2004; Marshall et al., 2020). Also, many other experiences may contribute to resilience in developmental outcomes for children from low SES backgrounds, including participation in enriching activities like art, music, and sports, that were not considered in our analysis. Although parental education may be conceptualized as a distal factor, it is considered here as a proximal measure that encompasses potential differences in the environment for children across the economic spectrum. For instance, it may be that higher parental education affords children with more opportunities for enriching learning and recreational activities, such as participating in music or sports (Guo and Mullan Harris, 2000; Bradley et al., 2001). Future studies should examine whether there are measurable differences in the quality and access of enriching activities that stimulate learning at all levels of parental education and whether participation in enriching activities for children among lower educated parents can be linked to positive developmental outcomes. However, there is likely not one single factor that will apply to all children with economic disadvantage in the same way. Thus, looking at the constellation of factors traveling with low SES helps identify malleable experiences that should be targeted synchronously by interventions. Last, cognitive and brain development is an on-going process and occurs reciprocally with many environmental proximal factors. The present cross-sectional study is unable to advance our understanding of this bi-directional process and warrants future longitudinal investigation.

## Conclusion

Within the large sample of children in the ABCD study, we conducted a descriptive investigation of 22 proximal measures of the lived experiences of children in association with SES and development. While our findings suggest that SES is an important determinant of developmental outcomes at age 9–10 years, in future studies within the ABCD cohort, we will be able to continue to examine the association of SES and proximal measures of the environment with brain development throughout adolescence. Beyond SES associations, we can examine the longitudinal influences of these latent ecologies and whether different patterns of relations among proximal measures emerge as stronger predictors during different stages of adolescence, i.e., will social support emerge as a stronger predictor of development in mid-adolescence?

## Data Availability Statement

The datasets presented in this study can be found in online repositories. The ABCD data repository grows and changes over time. The ABCD data used in this report came from DOI: 10.15154/1504041. The names of the repository/repositories and accession number(s) can be found below: NIH NDA https://nda.nih.gov/abcd.

## Ethics Statement

The studies involving human participants were reviewed and approved by Adolescent Brain Cognitive Development Study—central Institutional Review Board (cIRB) at the University of California, San Diego. Written informed consent to participate in this study was provided by the participants’ legal guardian/next of kin.

## Author Contributions

MG wrote the first draft of the manuscript. MG, CP, KU, TJ, WT, and ES edited and contributed text to the final manuscript. ES, TJ, KU, WT, and CP encouraged and contributed ideas for MG to investigate and develop the theoretical framework. CP supported MG in implementing the R code for the group factor analysis. CP generated the surface area effect size maps. MG performed all other analyses. WT supervised all analyses. All authors contributed to the article and approved the submitted version.

## Conflict of Interest

The authors declare that the research was conducted in the absence of any commercial or financial relationships that could be construed as a potential conflict of interest.
