# Supplementary material for: Positive Economic, Psychosocial, and Physiological Ecologies Predict Brain Structure and Cognitive Performance in 9–10-Year-Old Children
Source: Front Hum Neurosci. 2020 Oct 28;14:578822. doi: 10.3389/fnhum.2020.578822 (PMC7655980; doi:10.3389/fnhum.2020.578822)
Supplement: Supplementary file 1 [file Table_1.DOCX]

Supplementary Material

# Supplementary Tables

**Supplementary Table 1.** List of all measures with corresponding NDA variable names from the ABCD 2.0 dataset.

| **Group** | **Measure** | **ABCD Questionnaire** | **NDA variables and transformation** | **N Missing** | **N**  **Excluded** |
| --- | --- | --- | --- | --- | --- |
| **Economic Advantage** |  | Demographic Questionnaire | nda18$demo_comb_income_v2b  income-recoded:  2500 = Less than $5,000  8500 = $5,000 through $11,999  14000 = $12,000 through $15,999  20500 = $16,000 through $24,999  30000 = $25,000 through $34,999  42500 = $35,000 through $49,999  62500 = $50,000 through $74,999  87500 = $75,000 through $99,999  150000 = $100,000 through $199,999  250000 = $200,000 and greater  NA = Refuse to answer  NA = Don't know | 1,018 |  |
|  |  | 2017 Poverty Guidelines | Federal poverty guidelines <https://aspe.hhs.gov/2017-poverty-guidelines> | -- | -- |
|  | Household size | Demographic Questionnaire | nda18$demo_roster_p | 281 | 2 (>=50) |
|  | FPL | -- | Each participant assigned Federal poverty level (FPL) based on household size and 2017 Poverty Guidelines | 281 | 2 |
|  | Income-to-need | -- | (Income-recoded/FPL)*100 | 1213 |  |
| **Proximal Measures of Economic Security** | Food insecurity | Demographic Questionnaire | Recoded: No/Yes (0/1)  NA = Don’t know  demo_fam_exp1_v2b | 77 |  |
|  | Ability to pay bills | Demographic Questionnaire | Recoded: No/Yes (0/1)  NA = Don’t know  demo_fam_exp2_v2b or demo_fam_exp5_v2b | 48 |  |
|  | Housing Security | Demographic Questionnaire | Recoded: No/Yes (0/1)  NA = Don’t know  demo_fam_exp3_v2b or  demo_fam_exp4_v2b | 63 |  |
|  | Lack of Access to Medical/ Dental | Demographic Questionnaire | Recoded: No/Yes (0/1)  NA = Don’t know  demo_fam_exp6_v2b or  demo_fam_exp7_v2b | 52 |  |
| **Parental** | Parental Education | Demographic Questionnaire | high.educ (DEAP)  Responses were re-coded to numerical values corresponding to 1 (<High School); 2 (High School); 3 (Some college); 4 (B.A.); 4 (Post Graduate) | 17 |  |
|  | Youth Total Caregiver Warmth | Children's Report of Parental Behavioral Inventory | crpbi_ss_studycaregiver | 36 |  |
|  | Youth Parental Monitoring | Parental Monitoring Survey | Recoded:  1 = Strongly Disagree  2 = Disagree  3 = Neutral  4 = Agree  5 = Strongly Agree  NA = Don’t know  parental_monitoring_q1+ parental_monitoring_q2+ parental_monitoring_q3+ parental_monitoring_q4+ parental_monitoring_q5 | 25 |  |
|  | Duo Parent Households | Demographic Questionnaire | demo_prnt_prtnr_v2b | 126 |  |
| **School/ Community** | \| Youth  Neighborhood  Safety \| \| --- \| | Youth Neighborhood Safety/Crime Survey Modified from PhenX (NSC) | neighb_phenx | 26 |  |
|  | \| Youth Positive School Environment \| \| --- \| | School Risk and Protective Factors Survey | Recoded:  1 = No!  2 = no  3 = yes  4 = Yes!  NA = Don’t know  school_risk_phenx_2+ school_risk_phenx_3+ school_risk_phenx_4+ school_risk_phenx_5+ school_risk_phenx_6+  school_risk_phenx_7 | 28 |  |
|  | Youth School Engagement | School Risk and Protective Factors Survey | Recoded:  4 = No!  3 = no  2 = yes  1 = Yes!  NA = Don’t know  school_risk_phenx_15+  school_risk_phenx_17 | 27 |  |
| **ACEs** | Youth Family Conflict | Youth Family Environment Scale-Family Conflict Subscale Modified from PhenX (FES) | fes_y_ss_fc | 26 |  |
|  | History of Traumatic event | Parent Diagnostic Interview for DSM-5 (KSADS) Traumatic Events | Recoded: No/Yes (0/1)  NA = Don’t know  ksads_21_134_p | 185 |  |
|  | Parent Psychopathology | Family History Questionnaire & Adult Self Report (total problems score) | Recoded: No/Yes (0/1)  (z-score(asr_scr_totprob_t) +zscore(famhx_q9a_trouble_p or famhx_q9d_trouble _p ))/2 | 108 |  |
| **Perinatal** | \| Total Prenatal Conditions \|  \| \| --- \| --- \| | Developmental History Questionnaire | Recoded: No/Yes (0/1)  NA = Don’t know  devhx_10b_heavy_bleeding_p +  devhx_10c_eclampsia_p +  devhx_10d_gall_bladder_p +  devhx_10e_persist_proteinuria_p + devhx_10f_rubella_p + devhx_10g_severe_anemia_p + devhx_10h_urinary_infections_p + devhx_10i_diabetes_p + devhx_10j_high_blood_press_p + devhx_10k_problems_placenta_p + devhx_10l_accident_injury_p + devhx_10m_other_p | 712 |  |
|  | Planned Pregnancy | Developmental History Questionnaire | Recoded: No/Yes (0/1)  NA = Don’t know  devhx_6_pregnancy_planned_p | 267 |  |
|  | Maternal Age at Birth | Developmental History Questionnaire | devhx_3_age_at_birth_mother_p | 264 |  |
|  | History of Prenatal Substance Use | Developmental History Questionnaire | Recoded: No/Yes (0/1)  NA = Don’t know  (devhx_8_tobacco_p or  devhx_8_alcohol_p or devhx_8_marijuana_p or  devhx_8_coc_crack_p or  devhx_8_her_morph_p or  devhx_8_oxycont_p or  devhx_8_other_drugs) +  (devhx_9_tobacco_p or  devhx_9_alcohol_p or devhx_9_marijuana_p or  devhx_9_coc_crack_p or  devhx_9_her_morph_p or  devhx_9_oxycont_p or  devhx_9_other_drugs) | 730 |  |
|  | Gestational Age | Developmental History Questionnaire | 40 - devhx_12_weeks_premature_p | 173 |  |
|  | Birth Weight (kg) | Developmental History Questionnaire | (devhx_2_birth_wt_lbs_p + devhx_2b_birth_wt_oz_p) * 0.453592 | 525 | 2 (> 4.98 kg @ 35 weeks gestation) |
| **Physiological** | Sleep Hours | Youth Anthropometrics Modified From PhenX (ANT) survey | sleep_1_p  numerically recoded: 1 = less than 5 hours, 2 = 5-7 hours, 3 = 7-8 hours, 4 = 8-9 hours, 5 = 9 -11 hours | 6 |  |
|  | BMIz | Youth Anthropometrics Modified From PhenX (ANT) survey & SAS Program for the 2000 CDC Growth Charts^1^ | anthro_height_calc *2.54 (cm)  anthro_weight_calc *0.453592 (kg)  age  sex | 15 | 46  (BMIz < -4) |
| **Imaging** | Total Cortical Surface Area | sMRI Part 1 | smri_area_cort.desikan_total | 341 | 462 |
|  | Quality control for Freesurfer data | FreeSurfer QC | fsqc_qc | 337 | -- |
| **Cognition** | NIH Toolbox Total Computed Score Uncorrected | Youth NIH TB Summary Scores | nihtbx_fluidcomp_uncorrected | 249 |  |
| **Covariates** | MRI Serial number | MRI Info | mri_info_device.serial.number | 123 | -- |
|  | Race/ethnicity | DEAP | race.4level  demo_ethn_p  Recoded:  Hispanic (demo_ethn_p = 1)  White ((demo_ethn_p != 1 & race.4level  = “White”)  Black ((demo_ethn_p != 1 & race.4level  = “Black”)  Asian ((demo_ethn_p != 1 & race.4level  = “ Asian ”)  Other/Mixed ((demo_ethn_p != 1 & race.4level  = “ Other/Mixed ”) | 54 |  |
|  | Age | DEAP | age | 0 | -- |
|  | Sex | DEAP | sex  Recoded:  F (1)  M (0) | 4 | -- |
|  | Family | DEAP | rel_family_id | 0 | -- |
| ^1^Centers for Disease and Control and Prevention. A SAS Program for the 2000 CDC Growth Charts. Available at: https://www.cdc.gov/nccdphp/dnpao/growthcharts/resources/sas.htm. (Accessed: 19th August 2019) | | | | | |

**Supplementary Table 2.** Median latent factor loadings (95% CI) show consistent replication in latent factors across the reported robust GFA with the full sample, the robust GFAs for each split-half sample, the robust GFA with a sample that included singletons only, and the robust GFA with a sample randomly assigned only one participant per family. All robust GFAs were averaged across 10 GFA iterations.

|  | **Measure** | **Full Sample**  **N = 8158** | **Split-half sample 1**  **N = 3305** | **Split-half sample 2**  **N = 4079** | **Singletons Only**  **N = 4079** | **Random Sample for 1 participant per family**  **N = 6879** | |
| --- | --- | --- | --- | --- | --- | --- | --- |
| **Latent Factor 1** | Total Prenatal Conditions | -0.16 (-0.188, -0.1315) | -0.148 (-0.188, -0.108) | -0.164 (-0.2015, -0.126) | -0.156 (-0.1845, -0.1195) | -0.16 (-0.189, -0.133) | |
|  | Planned Pregnancy | 0.536 (0.507, 0.5615) | 0.509 (0.468, 0.5525) | 0.552 (0.5145, 0.5835) | 0.545 (0.515, 0.574) | 0.538 (0.51, 0.568) | |
|  | Maternal Age at Birth | 0.476 (0.4465, 0.5045) | 0.472 (0.44, 0.5115) | 0.477 (0.4295, 0.5175) | 0.474 (0.4425, 0.503) | 0.478 (0.44, 0.51) | |
|  | History of Prenatal Substance Use | -0.268 (-0.304, -0.2335) | -0.255 (-0.3075, -0.203) | -0.247 (-0.3075, -0.2045) | -0.266 (-0.305, -0.224) | -0.258 (-0.298, -0.215) | |
|  | Gestational Age | 0.022 (-0.002, 0.046) | 0.026 (-0.0115, 0.0615) | 0.019 (-0.012, 0.053) | 0.02 (-0.011, 0.0455) | 0.024 (-0.004, 0.051) | |
|  | Birth Weight | 0.054 (0.029, 0.078) | 0.066 (0.03, 0.102) | 0.04 (0.007, 0.0755) | 0.054 (0.0255, 0.081) | 0.05 (0.025, 0.08) | |
|  | Youth Family Conflict | -0.238 (-0.2625, -0.2065) | -0.204 (-0.239, -0.155) | -0.25 (-0.294, -0.21) | -0.233 (-0.2645, -0.1985) | -0.234 (-0.269, -0.206) | |
|  | History of Traumatic Event | -0.268 (-0.2925, -0.2435) | -0.24 (-0.2755, -0.205) | -0.276 (-0.312, -0.2425) | -0.268 (-0.297, -0.2415) | -0.264 (-0.292, -0.238) | |
|  | Parent psychopathology | -0.464 (-0.487, -0.442) | -0.45 (-0.484, -0.4165) | -0.454 (-0.4875, -0.42) | -0.463 (-0.487, -0.4355) | -0.458 (-0.482, -0.43) | |
|  | Highest Parent Education | 0.567 (0.5425, 0.5875) | 0.571 (0.5375, 0.6065) | 0.57 (0.539, 0.607) | 0.56 (0.535, 0.587) | 0.562 (0.539, 0.589) | |
|  | Youth Total Caregiver Acceptance | 0.144 (0.1105, 0.176) | 0.14 (0.083, 0.1865) | 0.126 (0.074, 0.173) | 0.145 (0.104, 0.1845) | 0.142 (0.109, 0.185) | |
|  | Youth Parental Monitoring | 0.21 (0.172, 0.2415) | 0.192 (0.142, 0.2375) | 0.202 (0.1555, 0.253) | 0.203 (0.1645, 0.244) | 0.208 (0.173, 0.245) | |
|  | Duo Parent Households | 0.395 (0.37, 0.4185) | 0.378 (0.3415, 0.413) | 0.421 (0.3875, 0.456) | 0.395 (0.368, 0.422) | 0.397 (0.371, 0.421) | |
|  | Sleep hours | 0.392 (0.3715, 0.414) | 0.428 (0.391, 0.456) | 0.376 (0.3465, 0.4075) | 0.4 (0.373, 0.4255) | 0.392 (0.368, 0.418) | |
|  | BMIz | -0.238 (-0.2615, -0.214) | -0.254 (-0.288, -0.2205) | -0.237 (-0.2705, -0.208) | -0.241 (-0.272, -0.2115) | -0.25 (-0.28, -0.227) | |
|  | Youth Neighborhood Safety | 0.318 (0.29, 0.344) | 0.281 (0.252, 0.319) | 0.312 (0.2805, 0.346) | 0.32 (0.2905, 0.3485) | 0.311 (0.281, 0.34) | |
|  | Youth Positive School Environment | 0.108 (0.073, 0.1435) | 0.078 (0.03, 0.121) | 0.108 (0.0615, 0.1525) | 0.101 (0.0625, 0.1395) | 0.102 (0.066, 0.148) | |
|  | Youth School Engagement | 0.111 (0.077, 0.139) | 0.076 (0.0365, 0.119) | 0.14 (0.098, 0.184) | 0.106 (0.073, 0.141) | 0.106 (0.073, 0.146) | |
|  | Food Security | 0.544 (0.522, 0.57) | 0.569 (0.5315, 0.6085) | 0.544 (0.5065, 0.581) | 0.546 (0.5215, 0.576) | 0.544 (0.522, 0.575) | |
|  | Ability to Pay Bills | 0.562 (0.538, 0.59) | 0.6 (0.562, 0.6355) | 0.555 (0.516, 0.588) | 0.571 (0.5455, 0.599) | 0.562 (0.536, 0.592) | |
|  | Housing Security | 0.556 (0.532, 0.5815) | 0.564 (0.5275, 0.596) | 0.569 (0.531, 0.605) | 0.56 (0.531, 0.5865) | 0.55 (0.521, 0.58) | |
|  | Access to Medical/Dental | 0.452 (0.43, 0.479) | 0.462 (0.4235, 0.495) | 0.45 (0.4135, 0.487) | 0.448 (0.42, 0.4745) | 0.448 (0.422, 0.471) | |
|  | | | | | | |  |
| **Latent Factor 2** | Total Prenatal Conditions | 0.018 (-0.0075, 0.0455) | 0 (0.0015, -0.0035) | 0.012 (-0.026, 0.0485) | 0.023 (0.06, -0.011) | 0.016 (-0.005, 0.043) | |
|  | Planned Pregnancy | -0.09 (-0.1235, -0.0465) | 0 (0, -0.0315) | -0.086 (-0.125, -0.0405) | -0.104 (-0.0595, -0.1545) | -0.074 (-0.129, -0.043) | |
|  | Maternal Age at Birth | -0.124 (-0.1745, -0.07) | 0 (0, -0.0485) | -0.138 (-0.192, -0.086) | -0.124 (-0.068, -0.1835) | -0.109 (-0.17, -0.047) | |
|  | History of Prenatal Substance Use | 0.034 (-0.0275, 0.102) | 0 (0.0085, 0) | 0.019 (-0.0675, 0.0885) | 0.06 (0.1285, -0.024) | 0.036 (-0.005, 0.111) | |
|  | Gestational Age | 0.002 (-0.015, 0.0255) | 0 (0, -0.007) | 0.015 (-0.013, 0.0485) | 0 (0.0255, -0.0245) | 0.004 (-0.016, 0.03) | |
|  | Birth Weight | -0.02 (-0.04, 0.002) | 0 (0, -0.012) | -0.02 (-0.047, 0.01) | -0.02 (0.0025, -0.0495) | -0.011 (-0.033, 0.012) | |
|  | Youth Family Conflict | -0.406 (-0.4395, -0.382) | -0.422 (-0.385, -0.4665) | -0.398 (-0.4365, -0.3525) | -0.409 (-0.3735, -0.4405) | -0.406 (-0.438, -0.378) | |
|  | History of Traumatic Event | 0.033 (0.003, 0.0745) | 0.012 (0.0535, -0.025) | 0.048 (-0.001, 0.0935) | 0.046 (0.085, 0.006) | 0.041 (0.01, 0.076) | |
|  | Parent psychopathology | -0.02 (-0.059, 0.0055) | -0.016 (0.025, -0.052) | -0.046 (-0.101, -0.0105) | -0.006 (0.035, -0.0475) | -0.02 (-0.054, 0.013) | |
|  | Highest Parent Education | -0.054 (-0.09, -0.0105) | 0.016 (0.0515, -0.018) | -0.081 (-0.129, -0.0285) | -0.037 (-0.0055, -0.072) | -0.041 (-0.074, -0.001) | |
|  | Youth Total Caregiver Acceptance | 0.578 (0.5485, 0.6035) | 0.597 (0.631, 0.5585) | 0.583 (0.5495, 0.624) | 0.574 (0.6035, 0.5435) | 0.591 (0.563, 0.62) | |
|  | Youth Parental Monitoring | 0.539 (0.5105, 0.569) | 0.566 (0.602, 0.5275) | 0.536 (0.496, 0.5705) | 0.538 (0.57, 0.5015) | 0.552 (0.521, 0.584) | |
|  | Duo Parent Households | -0.027 (-0.0615, 0.0045) | 0.012 (0.0615, -0.0445) | -0.047 (-0.095, 0) | -0.019 (0.017, -0.058) | -0.022 (-0.054, 0.016) | |
|  | Sleep hours | 0 (-0.0105, 0.0025) | 0 (0.0495, 0) | 0 (-0.044, 0) | 0 (5e-04, -0.014) | 0 (-0.01, 0.004) | |
|  | BMIz | 0 (0, 0.0425) | 0 (0.043, -0.0015) | 0 (0, 0.0595) | 0 (0.0435, 0) | 0 (0, 0.047) | |
|  | Youth Neighborhood Safety | 0.222 (0.191, 0.253) | 0.228 (0.2725, 0.186) | 0.224 (0.188, 0.278) | 0.226 (0.2585, 0.198) | 0.232 (0.202, 0.268) | |
|  | Youth Positive School Environment | 0.548 (0.5215, 0.572) | 0.535 (0.5705, 0.501) | 0.533 (0.4985, 0.573) | 0.534 (0.565, 0.499) | 0.546 (0.513, 0.573) | |
|  | Youth School Engagement | 0.439 (0.4095, 0.47) | 0.408 (0.4545, 0.367) | 0.43 (0.3915, 0.473) | 0.436 (0.471, 0.4005) | 0.436 (0.401, 0.469) | |
|  | Food Security | -0.09 (-0.1245, -0.053) | -0.063 (-0.023, -0.1235) | -0.095 (-0.1345, -0.0555) | -0.081 (-0.027, -0.124) | -0.102 (-0.142, -0.065) | |
|  | Ability to Pay Bills | -0.112 (-0.1425, -0.072) | -0.1 (-0.0645, -0.157) | -0.096 (-0.137, -0.051) | -0.1 (-0.037, -0.136) | -0.119 (-0.157, -0.093) | |
|  | Housing Security | -0.096 (-0.13, -0.056) | -0.079 (-0.0425, -0.133) | -0.084 (-0.13, -0.0405) | -0.079 (-0.032, -0.1225) | -0.099 (-0.135, -0.062) | |
|  | Access to Medical/Dental | -0.036 (-0.067, -0.0055) | -0.037 (0.001, -0.0855) | -0.034 (-0.0705, 0) | -0.028 (-0.0025, -0.0625) | -0.054 (-0.09, -0.016) | |
|  | | | | | | |  |
| **Latent Factor 3** | Total Prenatal Conditions | -0.428 (-0.4625, -0.391) | -0.428 (-0.369, -0.483) | -0.416 (-0.475, -0.3555) | -0.427 (-0.472, -0.377) | -0.426 (-0.469, -0.379) | |
|  | Planned Pregnancy | -0.064 (-0.0975, -0.0325) | -0.07 (-0.019, -0.117) | -0.057 (-0.1, -0.007) | -0.052 (-0.0935, -0.0095) | -0.062 (-0.098, -0.023) | |
|  | Maternal Age at Birth | -0.1 (-0.134, -0.0655) | -0.102 (-0.055, -0.145) | -0.102 (-0.149, -0.0485) | -0.106 (-0.1475, -0.0665) | -0.105 (-0.145, -0.07) | |
|  | History of Prenatal Substance Use | 0.054 (0.0095, 0.0945) | 0.062 (0.1225, 0.002) | 0.048 (-0.017, 0.114) | 0.059 (0.017, 0.1065) | 0.062 (0.012, 0.104) | |
|  | Gestational Age | 0.766 (0.744, 0.7855) | 0.756 (0.786, 0.7245) | 0.744 (0.7195, 0.773) | 0.768 (0.741, 0.795) | 0.768 (0.748, 0.787) | |
|  | Birth Weight | 0.747 (0.721, 0.7685) | 0.739 (0.773, 0.7045) | 0.722 (0.687, 0.758) | 0.744 (0.7175, 0.7705) | 0.746 (0.722, 0.777) | |
|  | Youth Family Conflict | 0 (0, 0) | 0 (0, 0) | 0 (0, 0) | 0 (0, 0) | 0 (0, 0) | |
|  | History of Traumatic Event | 0 (0, 0) | 0 (0, 0) | 0 (0, 0) | 0 (0, 0) | 0 (0, 0) | |
|  | Parent psychopathology | 0 (0, 0) | 0 (0, 0) | 0 (0, 0) | 0 (0, 0) | 0 (0, 0) | |
|  | Highest Parent Education | 0 (0, 0) | 0 (0, 0) | 0 (0, 0) | 0 (0, 0) | 0 (0, 0) | |
|  | Youth Total Caregiver Acceptance | 0 (0, 0) | 0 (0, 0) | 0 (0, 0) | 0 (0, 0) | 0 (0, 0) | |
|  | Youth Parental Monitoring | 0 (0, 0) | 0 (0, 0) | 0 (0, 0) | 0 (0, 0) | 0 (0, 0) | |
|  | Duo Parent Households | 0 (0, 0) | 0 (0, 0) | 0 (0, 0) | 0 (0, 0) | 0 (0, 0) | |
|  | Sleep hours | 0 (-0.023, 0.0245) | -0.014 (0.02, -0.0555) | 0.007 (-0.018, 0.0445) | 0.005 (-0.0225, 0.0305) | 0.006 (-0.018, 0.03) | |
|  | BMIz | 0.08 (0.0545, 0.1055) | 0.084 (0.146, 0) | 0.066 (0, 0.1075) | 0.086 (0.0585, 0.117) | 0.076 (0.047, 0.105) | |
|  | Youth Neighborhood Safety | 0 (-0.026, 0) | 0 (0, 0) | 0 (-0.047, 0) | 0 (-0.0415, 0) | 0 (-0.044, 0) | |
|  | Youth Positive School Environment | 0 (-0.011, 0) | 0 (0, 0) | 0 (-0.0305, 0) | 0 (-0.0235, 0) | 0 (-0.03, 0) | |
|  | Youth School Engagement | 0 (-0.027, 0) | 0 (0, 0) | 0 (-0.066, 0) | 0 (-0.0285, 0) | 0 (-0.041, 0) | |
|  | Food Security | 0 (0, 0) | 0 (0, 0) | 0 (0, 0) | 0 (0, 0) | 0 (0, 0) | |
|  | Ability to Pay Bills | 0 (0, 0) | 0 (0, 0) | 0 (0, 0) | 0 (0, 0) | 0 (0, 0) | |
|  | Housing Security | 0 (0, 0) | 0 (0, 0) | 0 (0, 0) | 0 (0, 0) | 0 (0, 0) | |
|  | Access to Medical/Dental | 0 (0, 0) | 0 (0, 0) | 0 (0, 0) | 0 (0, 0) | 0 (0, 0) | |

**Supplementary Table 3.** Results of log-likelihood tests comparing each model to a reduced model (fixed + random effects only) are shown, including *F (eds)* coefficients for smooth terms for the INR and latent factor 1, and standard beta coefficient values for all other linear terms. Separate models are shown in which the INR and latent factors predict total cognition scores.

|  |  | **Total Cognition Scores** | | | | | | | | | | | | | | | | | | | |
| --- | --- | --- | --- | --- | --- | --- | --- | --- | --- | --- | --- | --- | --- | --- | --- | --- | --- | --- | --- | --- | --- |
|  | |  | **Model 1: Income-to-needs** |  | **Model 2:**  **Latent Factor 1 + Income-to-needs** |  | | **Model 3:**  **Latent Factor 2 + Income-to-needs** | | | |  | | **Model 4:**  **Latent Factor 3 + Income-to-needs** | | |  | **Model 5:**  **Latent Factors 1, 2, & 3 + Income-to-needs** | | |  |
| *R^2^* | |  | 0.281 |  | 0.295 |  | | 0.282 | | | |  | | 0.287 | | |  | 0.302 | | |  |
| ∆*R^2^*_(Full – Reduced)_ | |  | 0.064 |  | 0.079 |  | | 0.066 | | | |  | | 0.071 | | |  | 0.086 | | |  |
| Chi-square  *p-value* | |  | 557.57  < 0.001 |  | 691.17  < 0.001 |  | | 576.89  < 0.001 | | | |  | | 597.95  <0.001 | | |  | 740.69  <0.001 | | |  |
| *s*(income-to-needs)  *F (edf)*  *p-value* | |  | 94.13 (6.43)  < 0.001 |  | 35.7 (6.4)  < 0.001 |  | | 94.51 (6.46)  < 0.001 | | | |  | | 95.61 (6.40)  < 0.001 | | |  | 36.9 (6.4)  < 0.001 | | |  |
| *s*(Latent Factor 1)  *F (edf)*  *p-value* | |  | -- |  | 40.6 (3.5)  < 0.001 |  | | -- | | | |  | | -- | | |  | 38.6 (3.4)  < 0.001 | | |  |
|  | |  | **Standardized betas (95% CI)** | | | | | | | | | | | | | | | | | |  |
| Latent Factor 2 | |  | -- |  | -- |  | 0.049 (0.027, 0.071) | | | |  | | -- | | |  | | 0.03 (0.02, 0.063) | | |  |
| Latent Factor 3 | |  | -- |  | -- |  | -- | | | |  | | 0.075 (0.052, 0.098) | | |  | | 0.073 (0.051, 0.096) | | |  |
| Age | |  | 0.002 (-0.015, 0.019) |  | 0.301 (0.281, 0.318) |  | 0.302 (0.28358, 0.32) | | | |  | | 0.301 (0.283, 0.319) | | |  | | 0.301 (0.283, 0.319) | | |  |
| Sex | |  | -0.908 (-0.943, -0.873) |  | 0.059 (0.029, 0.103) |  | 0.06 (0.02364, 0.097) | | | |  | | 0.071 (0.034, 0.108) | | |  | | 0.054 (0.017, 0.091) | | |  |
| Race-Ethnicity 1*^a^* | |  | -0.253 (-0.313, -0.194) |  | 0.095 (0.029, 0.16) |  | 0.101 (0.03558, 0.166) | | | |  | | 0.098 (0.033, 0.163) | | |  | | 0.100 (0.042, 0.171) | | |  |
| Race-Ethnicity 2*^a^* | |  | -0.624 (-0.691, -0.556) |  | 0.252 (0.212, 0.344) |  | 0.258 (0.19239, 0.323) | | | |  | | 0.276 (0.211, 0.341) | | |  | | 0.253 (0.194, 0.324) | | |  |
| Race-Ethnicity 3*^a^* | |  | -0.206 (-0.354, -0.058) |  | -0.30 (-0.459, -0.254) |  | -0.314 (-0.41628, -0.212) | | | |  | | -0.36 (-0.462, -0.258) | | |  | | -0.309 (-0.425, -0.222) | | |  |
| Race-Ethnicity 4*^a^* | |  | -0.237 (-0.302, -0.172) |  | -0.40 (-0.548, -0.371) |  | -0.408 (-0.50, -0.32) | | | |  | | -0.465 (-0.553, -0.377) | | |  | | -0.414 (-0.505, -0.33) | | |  |
| Note: sex was dummy coded as 0 = Male and 1 = Female.  ^a^Reference group =Race-Ethnicity 5. | | | | | | | | |  |  | | | | |  | | | |  |  |  |

**Supplementary Table 3.** Results of models predicting total cortical surface area. A log-likelihood test compared the change in R^2^ between each model and the null model (fixed + random effects only).

|  |  | **Total Cortical Surface Area** | | | | | | | | | | | | | | | | | | | | |
| --- | --- | --- | --- | --- | --- | --- | --- | --- | --- | --- | --- | --- | --- | --- | --- | --- | --- | --- | --- | --- | --- | --- |
|  | | |  | **Model 1: Income-to-needs** |  | **Model 2:**  **Latent Factor 1 + Income-to-needs** |  | | **Model 3:**  **Latent Factor 2 + Income-to-needs** | | | |  | | **Model 4:**  **Latent Factor 3 + Income-to-needs** | | |  | **Model 5:**  **Latent Factors 1, 2, & 3 + Income-to-needs** | | |  |
| *R^2^* | | |  | 0.276 |  | 0.29 |  | | 0.289 | | | |  | | 0.289 | | |  | 0.303 | | |  |
| ∆*R^2^*_(Full – Reduced)_ | | |  | 0.0117 |  | 0.014 |  | | 0.0124 | | | |  | | 0.027 | | |  | 0.030 | | |  |
| Chi-square  *p-value* | | |  | 120.66  < 0.001 |  | 162.68  < 0.001 |  | | 130.46  < 0.001 | | | |  | | 236.03  <0.001 | | |  | 282.52  <0.001 | | |  |
| *s*(income-to-needs)  *F (edf)*  *p-value* | | |  | 34.82 (3.77)  < 0.001 |  | 13.17 (3.07)  < 0.001 |  | | 34.73 (3.81)  < 0.001 | | | |  | | 35.22 (3.71)  < 0.001 | | |  | 13.91 (3.10)  < 0.001 | | |  |
|  | | |  | **Standardized betas (95% CI)** | | | | | | | | | | | | | | | | | |  |
| Latent Factor 1 | | |  | -- |  | 0.086 (0.06, 0.112) |  | -- | | | |  | | -- | | |  | | 0.081 (0.055, 0.107) | | |  |
| Latent Factor 2 | | |  | -- |  | -- |  | 0.033 (0.012, 0.053) | | | |  | | -- | | |  | | 0.027 (0.007, 0.047) | | |  |
| Latent Factor 3 | | |  | -- |  | -- |  | -- | | | |  | | 0.123 (0.101, 0.145) | | |  | | 0.121 (0.099, 0.143) | | |  |
| Age | | |  | 0.003 (-0.014, 0.02) |  | 0.004 (-0.012, 0.021) |  | 0.001 (-0.016, 0.018) | | | |  | | 0.004 (-0.012, 0.021) | | |  | | 0.005 (-0.012, 0.021) | | |  |
| Sex | | |  | -0.905 (-0.94, -0.87) |  | -0.909 (-0.943, -0.874) |  | -0.914 (-0.949, -0.879) | | | |  | | -0.898 (-0.932, -0.863) | | |  | | -0.908 (-0.943, -0.873) | | |  |
| Race-Ethnicity 1*^a^* | | |  | -0.129 (-0.193, -0.066) |  | -0.126 (-0.189, -0.063) |  | -0.128 (-0.191, -0.065) | | | |  | | -0.123 (-0.186, -0.06) | | |  | | -0.12 (-0.182, -0.057) | | |  |
| Race-Ethnicity 2*^a^* | | |  | 0.329 (0.266, 0.393) |  | 0.318 (0.254, 0.381) |  | 0.332 (0.268, 0.395) | | | |  | | 0.326 (0.263, 0.389) | | |  | | 0.317 (0.254, 0.38) | | |  |
| Race-Ethnicity 3*^a^* | | |  | -0.104 (-0.203, -0.005) |  | -0.079 (-0.178, 0.019) |  | -0.107 (-0.206, -0.009) | | | |  | | -0.108 (-0.206, -0.01) | | |  | | -0.087 (-0.185, 0.011) | | |  |
| Race-Ethnicity 4*^a^* | | |  | -0.258 (-0.343, -0.172) |  | -0.229 (-0.315, -0.143) |  | -0.259 (-0.345, -0.174) | | | |  | | -0.261 (-0.347, -0.176) | | |  | | -0.236 (-0.321, -0.15) | | |  |
| Note: sex was dummy coded as 0 = Male and 1 = Female.  ^a^Reference group =Race-Ethnicity 5. | | | | | | | | | |  |  | | | | |  | | | |  |  |  |

# Supplementary Figures

**
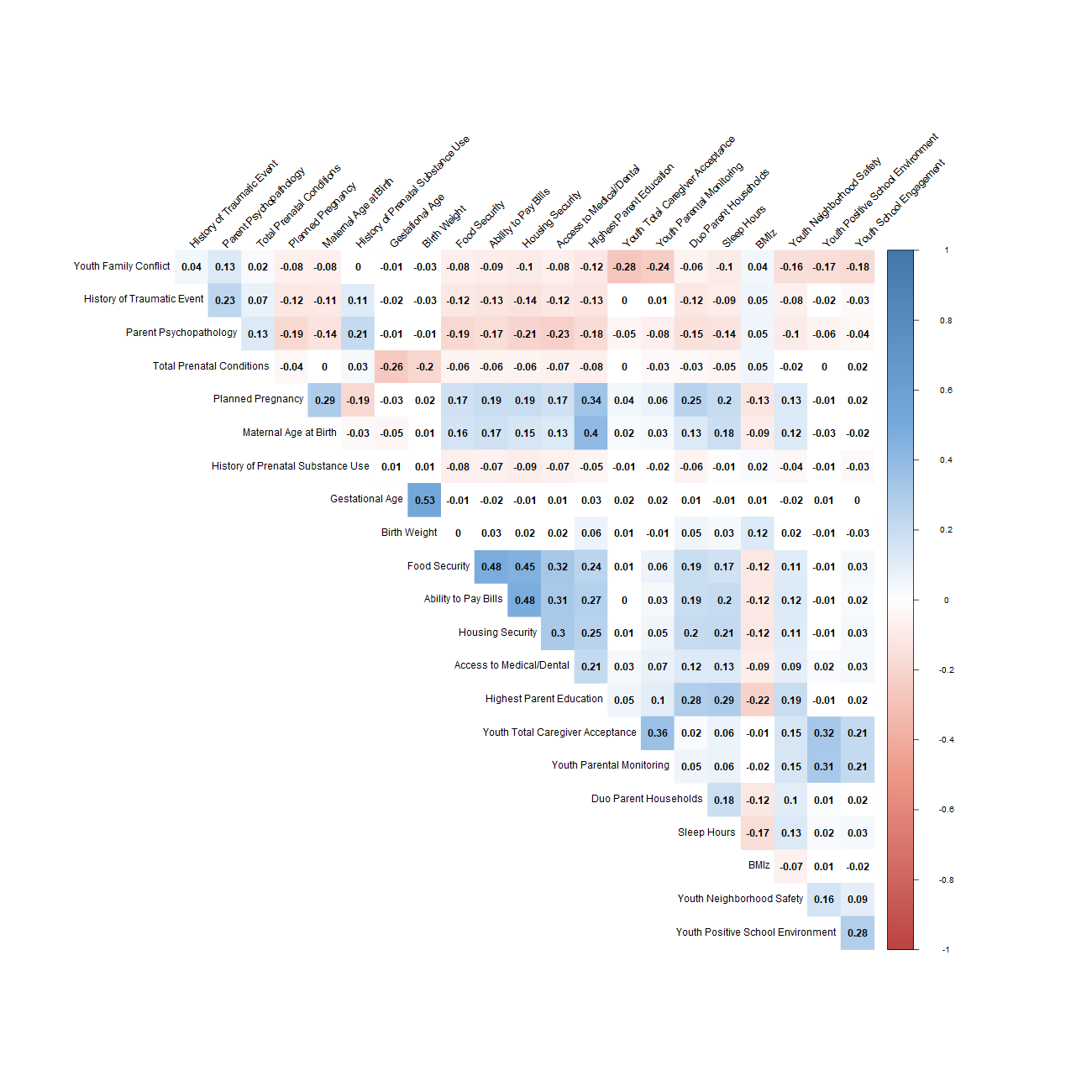
**

**Supplementary Figure 1.**  Spearman correlation matrix showing the correlations between the 22 proximal measures encompassing economic, social, physiological and perinatal ecologies.

**
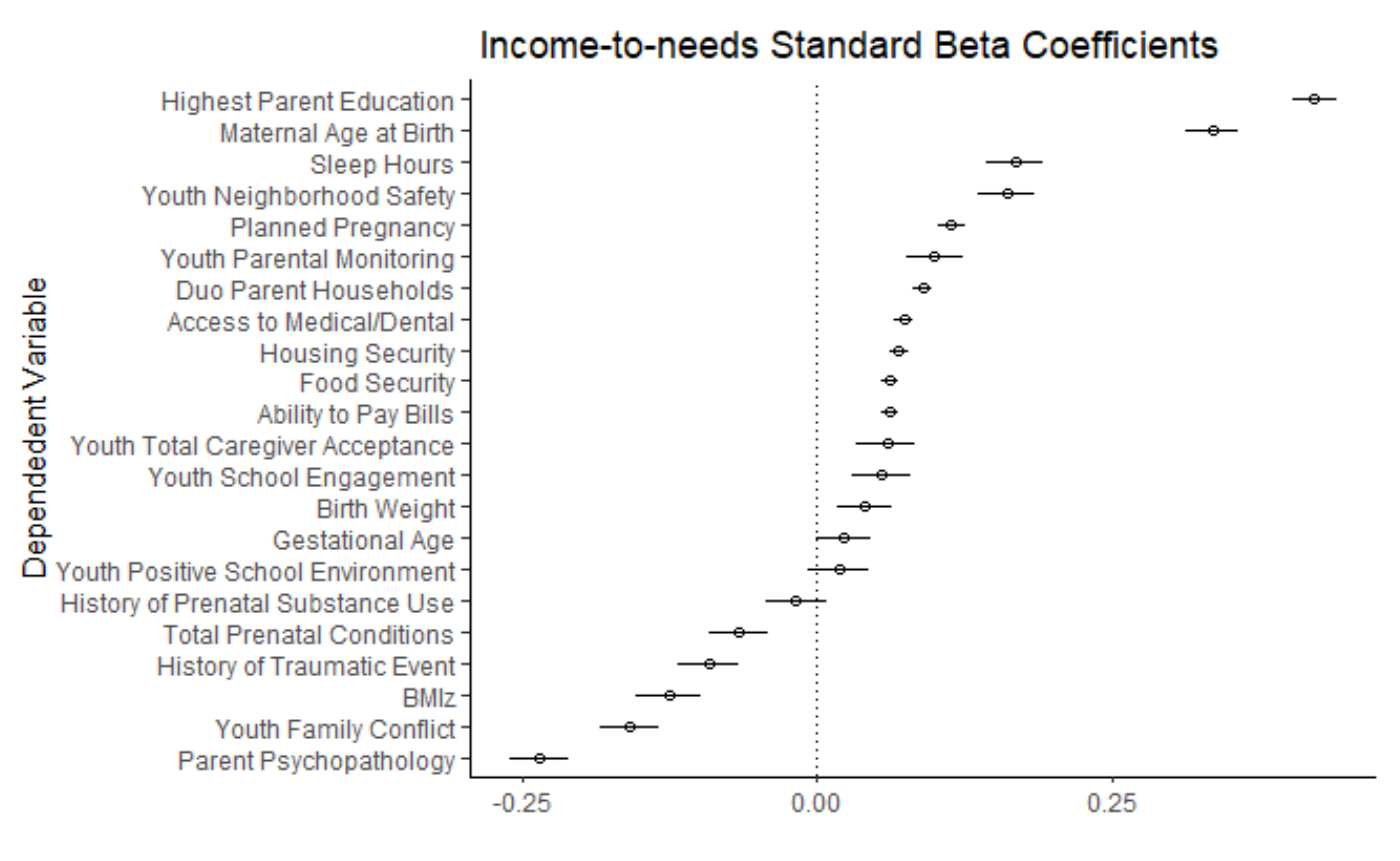
Supplementary Figure 2.** Plot shows the standard beta coefficients showing the strength of the association between the income-to-needs ratio and each proximal measure included in the GFA analysis (all models controlled for age, sex, race-ethnicity, scanner identification and family identification).


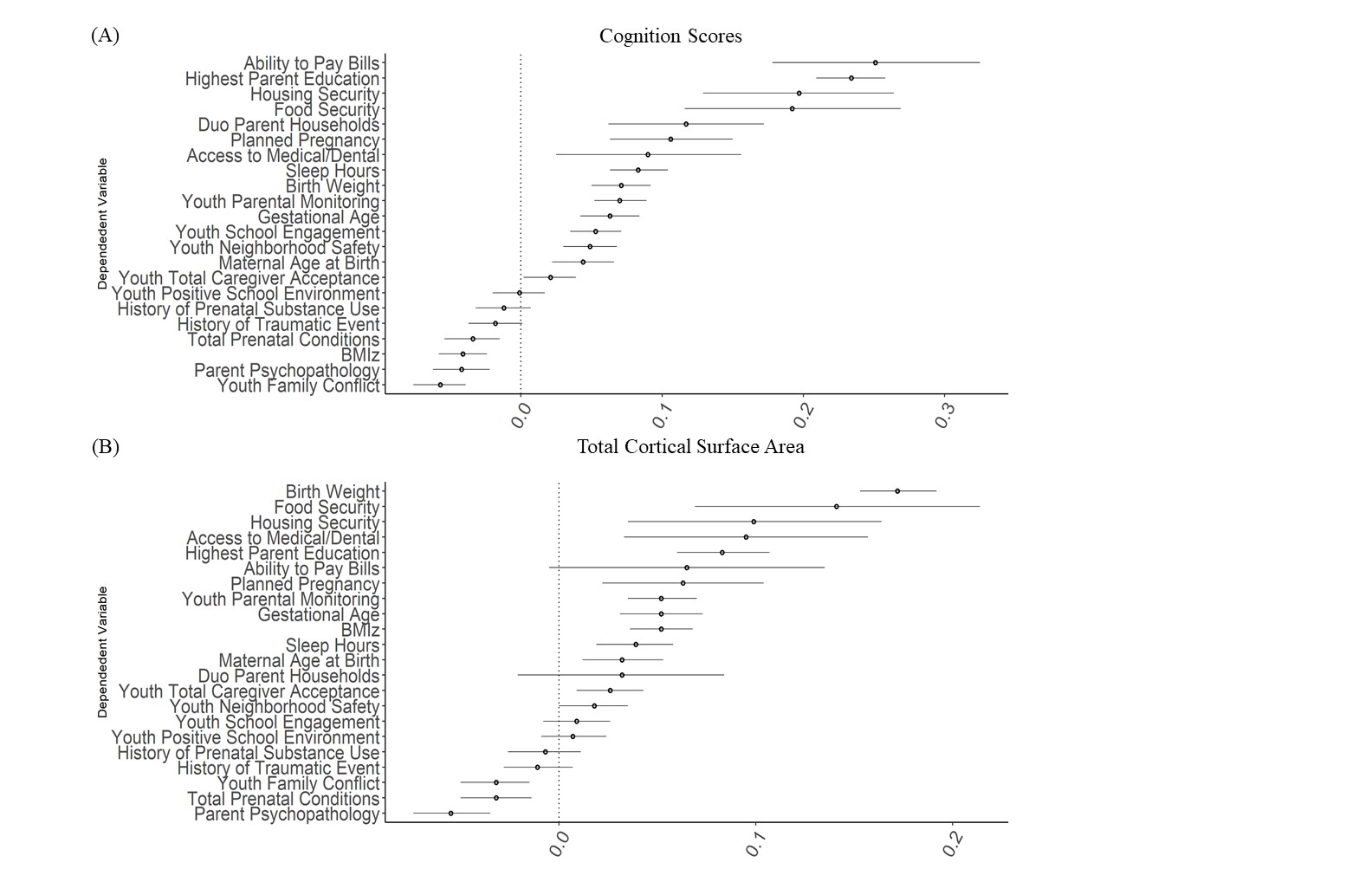


**Supplementary Figure 3.** A post-hoc analysis was done to evaluate the individual associations between each proximal measure and the developmental measures. The plots show the standard beta coefficients showing the strength of the association between each proximal measure included in the GFA analysis with (A) total cognition scores, and (B) total cortical surface area, all models included covariates of income-to-needs, age, sex, race-ethnicity, scanner identification and family identification).

**
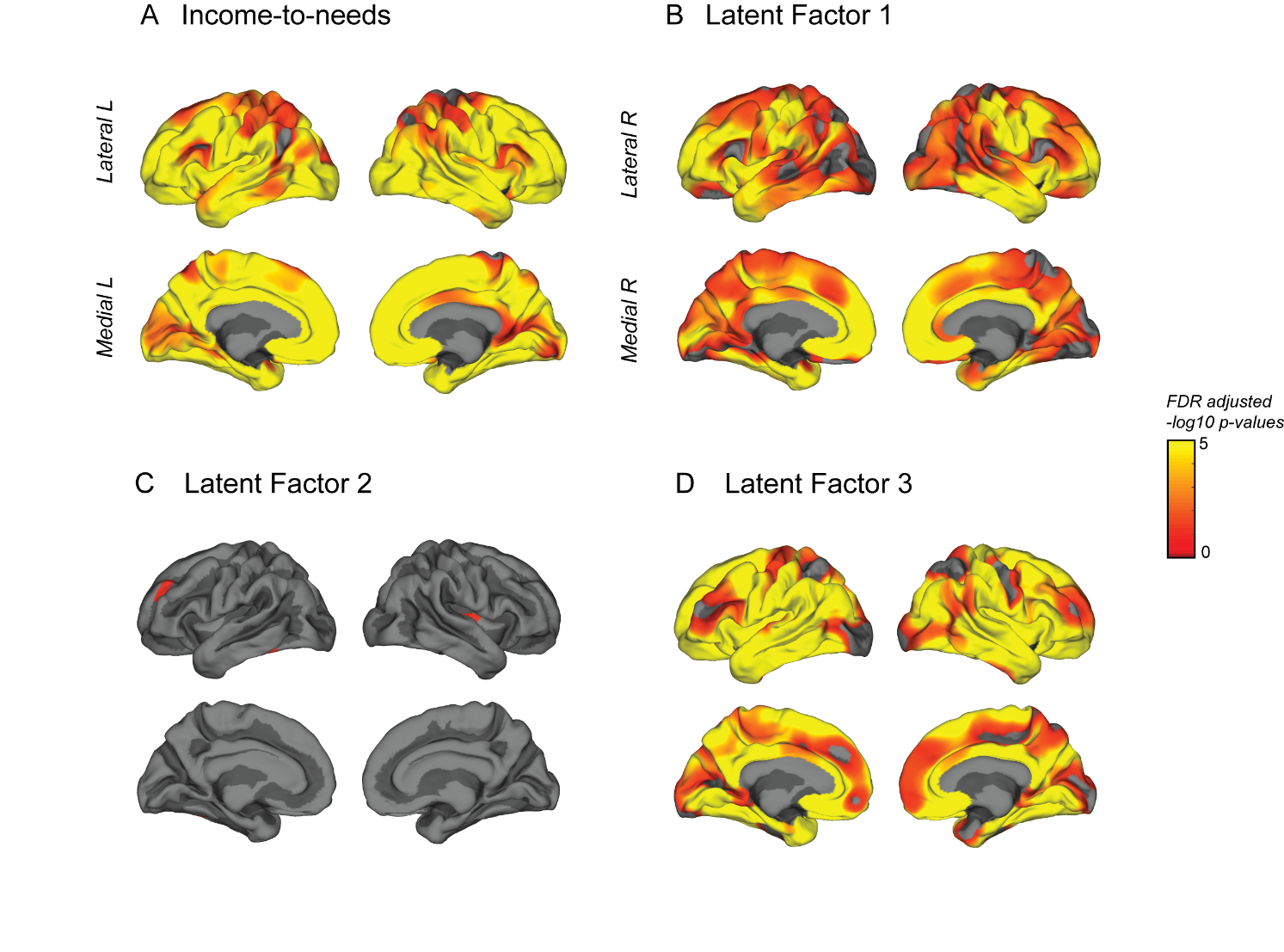
**

**Supplementary Figure 4.**  Mass univariate thresholded vertexwise p-values, adjusted for a false discovery rate (FDR) of 5%, predicting surface area from each independent variable, (a) income-to-needs, (b) latent factor 1, (c) latent factor 2, (d) latent factor 3 at each vertex, controlling for age, sex, race/ethnicity, and scanner. To account for the genetic relatedness across the sample, we selected only one member from each family to be included in the analysis. This created an N of 6954. Maps b-d included income-to-needs and the other latent factors as additional covariates such that these maps show the unique contribution of each latent factor and surface area.
